# Recent Advancements in Drug Delivery Across the Blood–Brain Barrier in Amyotrophic Lateral Sclerosis (ALS)

**DOI:** 10.1002/cns.71078

**Published:** 2026-09-11

**Authors:** Nitesh Sanghai, Kaitlyn Pierce, Nidhi Sharma, Sasha Leggett, Tara Chand Yadav, Pegah Masrori, Devi Atukorallaya, Senthilnathan Palaniyandi, Paul C. Marcogliese, Geoffrey K. Tranmer

**Affiliations:** ^1^ College of Pharmacy, Rady Faculty of Health Sciences University of Manitoba Winnipeg Manitoba Canada; ^2^ Cellular and Molecular Medicine Program Johns Hopkins University School of Medicine Baltimore Maryland USA; ^3^ Department of Neurobiology University of Chicago Chicago Illinois USA; ^4^ Division of Hematology and Medical Oncology, Department of Medicine, Ellis Fischel Cancer Center University of Missouri Columbia Missouri USA; ^5^ Department of Neurology Cliniques Universitaires Saint‐Luc Brussels Belgium; ^6^ Institute of Neuroscience UCLouvain Brussels Belgium; ^7^ Department of Oral Biology, Dr. Gerald Niznick College of Dentistry, Rady Faculty of Health Sciences University of Manitoba Winnipeg Manitoba Canada; ^8^ Department of Biochemistry and Medical Genetics, Rady Faculty of Health Sciences University of Manitoba Winnipeg Manitoba Canada

## Abstract

**Background:**

Amyotrophic lateral sclerosis (ALS) is a progressive and fatal motor neurodegenerative disease with limited therapeutic options. The blood–brain barrier (BBB) and blood‐spinal cord barrier (BSCB) present major obstacles to central nervous system (CNS) drug delivery, restricting the effectiveness of many potential therapies. Increasing evidence suggests that BBB and BSCB dysfunction are not only barriers to treatment but also important contributors to ALS pathophysiology.

**Objective:**

To examine current evidence regarding BBB and BSCB dysfunction in ALS and evaluate the implications of stage‐dependent barrier alterations for CNS drug delivery and therapeutic outcomes.

**Methods:**

Recent mechanistic, pathological, preclinical, and clinical studies examining BBB and BSCB alterations in ALS were reviewed. Evidence on tight junction disorganization, endothelial dysfunction, vascular leakage, altered transporter activity, and emerging therapeutic and drug delivery strategies was analyzed.

**Results:**

Converging evidence indicates that BBB and BSCB dysfunction are intrinsic and progressive features of ALS pathophysiology rather than passive consequences of neurodegeneration. Barrier impairment emerges early in the disease course and evolves across clinical stages. Disruption of barrier integrity may increase motor neuron vulnerability while modulating CNS drug exposure. Emerging strategies that bypass, exploit, or restore barrier function hold promise for enhancing CNS bioavailability and improving therapeutic outcomes.

**Conclusions:**

Improved understanding of barrier alterations may facilitate development of more effective CNS‐targeted therapies and improve therapeutic outcomes in ALS patients.

## Highlights and Perspective

- The BBB is a highly selective, protective lipophilic membrane composed of endothelial cells, astrocytes, and pericytes, within the neurovascular unit. It protects the CNS from toxins, pathogens, and immune signals, while tightly regulating the transport of ions, molecules, and cells essential for neuronal homeostasis and function.
- The BBB is crucial in neurological diseases. Alterations in BBB integrity, including increased permeability, endothelial injury, and dysregulated transport, enable the influx of neurotoxic and inflammatory mediators into the CNS, compromising the tightly regulated homeostasis essential for neuronal survival and leading to inflammation, edema, and irreversible tissue damage.
- The BBB represents a major pharmacokinetic barrier in neurotherapeutics: Over 98% small‐molecule drugs and nearly all large‐molecule therapeutics fail to adequately penetrate the CNS. Efflux transporters and tight junctions further restrict drug accumulation, resulting in limited bioavailability of potentially neuroprotective agents.
- Emerging strategies to enhance CNS drug delivery increasingly focus on targeted and minimally invasive approaches, including focused ultrasound (FUS), engineered nanocarriers, and intrathecal or intracerebroventricular administration. When combined with advanced imaging modalities, they allow for spatially and temporally controlled modulation of BBB permeability, improving drug delivery while limiting systemic exposure.
- BBB and BSCB dysfunction are increasingly recognized as features of ALS pathology that can emerge early in the disease course and may contribute to neurodegeneration through impaired barrier integrity and altered CNS homeostasis.
- Importantly, increased barrier permeability in ALS does not necessarily translate into improved drug delivery. Compensatory mechanisms, including altered transporter activity and heterogeneous vascular pathology, may limit effective drug penetration and distribution within target regions.
- Identifying robust and noninvasive markers of BBB and BSCB alterations is therefore essential to stratify patients and guide therapeutic strategies. Such markers may help define clinical disease stages and identify subgroups in whom barrier alterations differentially impact CNS drug exposure and treatment response.
- Future ALS drug discovery should prioritize BBB/BSCB‐permeable, CNS‐optimized small molecules with favorable PK properties and resistance to efflux transporters to improve brain and spinal cord drug exposure.

## Graphical abstract

Progressive BBB/BSCB dysfunction is increasingly recognized as both a pathological feature and a therapeutic barrier in ALS. Novel delivery technologies and barrier‐restoration strategies may enhance CNS drug exposure, improve the efficacy of disease‐modifying therapies, and advance ALS treatment.

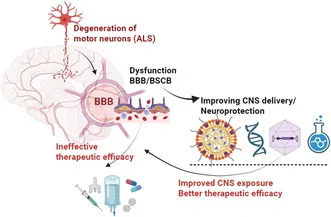

**Abbreviations**
AAV: adeno‐associated virus
ALS: amyotrophic lateral sclerosis
APC: activated protein C
ASO: antisense oligonucleotide
BBB: blood–brain barrier
BSCB: blood‐spinal cord barrier
CAG: cytomegalovirus/chicken β‐actin
ChAT: choline acetyltransferase
CMV: cytomegalovirus
CNS: central nervous system
CSF: cerebrospinal fluid
DEG: deferasirox
DFX: differentially expressed gene
EC: endothelial cell
EDR: edaravone
epSPC: ependymal stem/progenitor cell
FTD: frontotemporal dementia
FUS: focused ultrasound
GBM: graphene‐based material
GFAP: glial fibrillary acidic protein
GSH: glutathione
GSHE: glutathione monoethyl ester
HIFU: high‐intensity focused ultrasound
hSyn1: human synapsin‐1
IBEC: induced pluripotent stem cell‐derived brain endothelial‐like cell
IGF‐11: insulin‐like growth factor 1
IgG: immunoglobulin G
LEP: leptin
LIPUS: low‐intensity pulsed ultrasound
LNS: lipid nanosystem
LPS: lipopolysaccharide
MB: microbubble
MBs: microbubbles
MEF: microvessel‐enriched fraction
miRNA: microRNA
MS: multiple sclerosis
MTDLs: multi‐target‐directed ligands
N2B: nose‐to‐brain
NDD: neurodegenerative disorders
PEG: polyethylene glycol
PET: positron emission tomography
P‐gp: P‐glycoprotein
PIO: pioglitazone
PLA–PEG: poly(lactic acid)‐poly(ethylene glycol)
PLGA: poly(lactic‐co‐glycolic acid)
RARβ: retinoic acid receptor‐β
ROS: reactive oxygen species
RZ: Riluzole
SG: stress granule
SNAP‐25: synaptosomal‐associated protein 25
SOD: superoxide dismutase
TDP‐43: TAR DNA protein‐43
TJ: tight junction
TLR9: toll‐like receptor 9
TRPV4: transient receptor potential vanilloid 4
WT: wild‐type
ZO‐1: zonula occludens‐1

## 1 Questions Needing Further Investigation

The BBB serves as a protective interface for the CNS but remains a principal obstacle in neurotherapeutic development. In ALS, progressive and age‐dependent alterations in BBB and BSCB integrity and permeability raise a key therapeutic question: When and in which subset of ALS patients should drug delivery be optimized to maximize therapeutic efficacy? Variability in barrier integrity suggests that CNS drug exposure is neither static nor uniform across patients but instead evolves over the course of the disease.

Addressing this challenge requires a BBB‐aware clinical trial design that integrates disease staging, patient heterogeneity, and pharmacokinetic profiling. Parallel advances in experimental models that accurately recapitulate ALS‐specific barrier dysfunction will be essential. Ultimately, a more precise understanding of barrier dynamics may enable stage‐adapted therapeutic strategies and improve the success of CNS‐targeted interventions in ALS.

## 2 Introduction

The healthy central nervous system (CNS) is protected by the blood–brain barrier (BBB), a highly selective and dynamic physiological interface composed of endothelial cells (ECs), pericytes, and astrocyte end‐feet within the neurovascular unit. This semi‐permeable barrier shields the brain from xenobiotics, pathogens, and peripheral inflammatory signals while tightly regulating molecular trafficking, allowing the entry of essential nutrients but excluding the vast majority of therapeutic compounds. It is estimated that over 98% of small‐molecule drugs [1] and nearly all large molecules (> 400–600 Da) [2] fail to penetrate the BBB, significantly limiting CNS drug delivery.

This restrictive property represents a major challenge for the delivery of neurotherapeutics in neurological disorders such as amyotrophic lateral sclerosis (ALS). ALS is a fatal, progressive motor neurodegenerative disease characterized by the degeneration of upper and lower motor neurons in the brain and spinal cord. It is a highly complex, heterogeneous, and multifactorial disorder with an incompletely understood etiology [3]. Despite being first described by Charcot in 1869, therapeutic progress remains limited, with only two small organic molecules approved by the FDA, Edaravone (EDR) and Riluzole (RZ), widely used, providing modest clinical benefits and extension of life span in patients with ALS [4]. Further, the recent accelerated approval of tofersen, an antisense oligonucleotide (ASO), was based on reductions in neurofilament light chain (NfL), a nonspecific surrogate biomarker of neuroaxonal injury [5].

A key obstacle in ALS drug development is the combined barrier function of the BBB and the blood–spinal cord barrier (BSCB), which restricts drug access to motor neurons in both the brain and spinal cord. In addition to limited passive permeability, active efflux mediated by multidrug transporters further reduces CNS drug concentrations, often preventing compounds from reaching therapeutic levels at target sites. Consequently, failure in therapeutic development may partly reflect inadequate CNS exposure rather than a lack of biological efficacy.

To address these challenges, substantial efforts have been directed toward the development of advanced drug delivery strategies aimed at improving CNS bioavailability while minimizing systemic toxicity. These include intrathecal and intracerebroventricular delivery, focused ultrasound–mediated BBB modulation, receptor‐mediated transcytosis, carrier‐based systems such as nanoparticles, and viral vector–based approaches. Collectively, these strategies seek to bypass, exploit, or transiently modulate barrier functions to enhance targeted delivery of neuroprotective agents.

This review first examines early evidence of BBB and BSCB disruption in both preclinical models and patients with ALS. It then summarizes recent advances in BBB‐targeted drug delivery technologies, including ultrasound‐assisted approaches. Subsequently, we discuss emerging strategies such as Viral Vector‐Mediated Delivery in experimental ALS models. Finally, we highlight innovative approaches to optimize the CNS bioavailability of therapeutic agents, including EDR, with the aim of improving clinical efficacy for ALS therapeutics.

## 3 Why the Blood–Brain Barrier Is Important in ALS

The BBB and BSCB are essential interfaces that preserve CNS homeostasis by regulating the exchange of nutrients, metabolites, immune cells, and potentially harmful circulating factors between the blood and neural tissue. In neurological diseases, disruption of these barriers can profoundly alter the CNS microenvironment, permitting the entry of blood‐derived proteins, inflammatory mediators, immune cells, and toxic molecules that are normally excluded. Such changes may amplify neuroinflammation, oxidative stress, edema, and neuronal vulnerability, thereby contributing to disease initiation or progression rather than simply reflecting secondary tissue damage [6, 7, 8].

In ALS, the relevance of BBB and BSCB dysfunction is particularly important because motor neurons are located in regions that depend on tightly regulated vascular support, including the motor cortex, brainstem, and spinal cord anterior horns. Experimental studies in mutant SOD1 (mSOD1) models have shown that BSCB disruption can occur before overt motor neuron degeneration and before the appearance of clinical symptoms. Early vascular abnormalities may include reduced tight junction protein expression, endothelial injury, basement membrane alterations, pericyte dysfunction, IgG and hemosiderin deposition, and reduced spinal cord perfusion, especially in the anterior horn [9, 10, 11]. These observations suggest that barrier dysfunction may act as an upstream contributor to motor neuron injury, rather than merely a late consequence of neurodegeneration.

The timing of BBB and BSCB alterations is therefore central to understanding ALS pathogenesis. Presymptomatic or early‐stage barrier disruption may expose vulnerable motor neurons to blood‐derived neurotoxic factors, oxidative stress, and inflammatory signals. As disease progresses, barrier pathology may become more extensive and heterogeneous, interacting with microglial activation, astrocytosis, metabolic stress, and impaired vascular regulation [10, 11, 12]. This stage‐dependent evolution has important therapeutic implications: treatments delivered too late may fail not only because motor neurons are already irreversibly damaged, but also because the barrier environment has become structurally and functionally altered.

BBB and BSCB integrity also directly influence drug efficacy in ALS. Although barrier leakage might appear to facilitate drug entry, increased permeability does not necessarily translate into effective therapeutic delivery. Drug penetration remains constrained by regional heterogeneity, altered transporter activity, efflux mechanisms such as P‐glycoprotein, impaired perfusion, and inadequate distribution to target motor neuron compartments [12, 13]. Therefore, ALS drug failure may partly reflect insufficient CNS exposure, even when a compound shows biological activity in vitro or in peripheral compartments.

**FIGURE 1:**
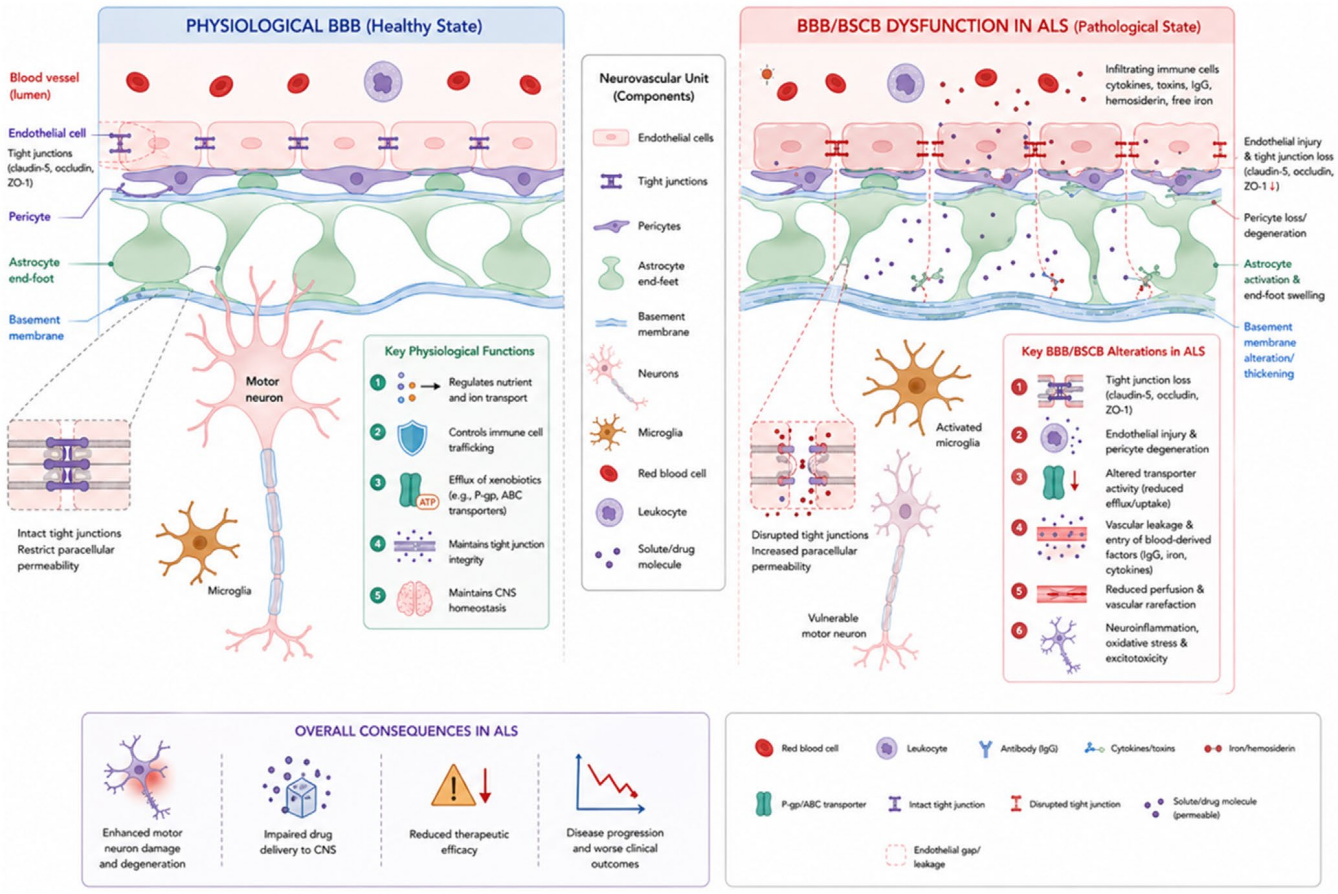
Schematic of the structure and function of the blood–brain barrier (BBB) and its disruption in amyotrophic lateral sclerosis (ALS). Under physiological conditions, the BBB is formed by tightly connected endothelial cells, supported by pericytes, astrocytic end‐feet, and the basement membrane within the neurovascular unit, ensuring controlled transport of nutrients, immune surveillance, and exclusion of xenobiotics. In ALS, this organization is compromised, with loss of tight junction integrity, endothelial and pericyte dysfunction, altered transporter activity, and increased permeability. These changes permit the entry of blood‐derived factors and immune mediators into the central nervous system, contributing to neuroinflammation and increased motor neuron vulnerability.

These considerations highlight the need to incorporate BBB‐ and BSCB‐aware strategies into ALS therapeutic development. Biomarkers of barrier dysfunction, including imaging‐based measures, blood or CSF indicators of vascular leakage, and molecular signatures of endothelial injury, could help stratify patients according to disease stage and vascular phenotype [7, 14, 15]. Such stratification may identify patients most likely to benefit from barrier‐stabilizing therapies, transporter‐targeted approaches, or advanced delivery methods such as focused ultrasound, intrathecal administration, receptor‐mediated transport, nanocarriers, or viral vectors [16, 17, 18, 19]. Ultimately, understanding when and how BBB/BSCB dysfunction occurs in ALS may improve patient selection, optimize treatment timing, and increase the likelihood that neurotherapeutics reach their intended CNS targets (Figure 1).

## 4 Evidence of Blood–Brain Barrier Disruption in Preclinical ALS Models

In 2025, two landmark studies delineated the role of TDP‐43 (TAR DNA‐binding protein 43) in BBB disruption in neurodegenerative disorders (NDs). Omar et al. [20] showed that nuclear levels of TDP‐43 in endothelial cells (ECs) change and are linked to BBB disruption in NDs. ECs are central to BBB integrity but undergo structural and functional deterioration across neurodegenerative conditions. They are sealed by proteins within tight junctions, including claudin‐5, occludin, and zonula occludens, creating a barrier that limits the diffusion of hydrophilic molecules. ECs control nutrient transport, for example, glucose via Glucose Transporter Type 1 (GLUT1) and toxin removal (e.g., via P‐glycoprotein), interacting with astrocytes and pericytes of the neurovascular unit that strengthen the barrier. EC deterioration is a shared feature of stroke, ALS, Alzheimer's disease (AD), multiple sclerosis (MS), and a weakened BBB [21].

In their study, they identified a proinflammatory, barrier‐compromised endothelial subtype in NDs, including AD, ALS, and frontotemporal dementia (FTD). In these ECs, nuclear TDP‐43 levels were found to be reduced. This is sufficient to increase the canonical p65 subunit of nuclear factor kappa‐light‐chain‐enhancer of activated B cells (NF‐κB), a key regulator of cellular inflammation and immune responses, and to disrupt Wnt/β‐catenin signaling. Therefore, a deeper understanding of the molecular mechanisms underlying these ECs may facilitate the identification of early vascular biomarkers and the development of strategies to mitigate BBB and vascular dysfunction in neurodegeneration.

After identifying reduced TDP‐43 expression in a reactive ECs subtype in post‐mortem brains from donors with ALS, AD, or FTD [20], Cheemala et al. [22] hypothesized that the loss of nuclear TDP‐43 in ECs contributes to BBB dysfunction. To investigate this, tomato lectin 488 dye was administered via retro‐orbital injection into the brains of *Tardbp* ^*G348C/+*^ mice, an ALS‐FTD model with reduced nuclear TDP‐43, allowing systemic circulation and assessment of vascular integrity. BBB leakage was observed only in 10–11‐month‐old mice, but not in 3‐month‐old *Tardbp* ^*G348C/+*^ mice, compared to age‐matched controls, indicating an age‐related increase in BBB dysfunction. These older mice also exhibited increased astrocytosis and microgliosis, along with elevated phospho‐Tau levels, consistent with neuronal damage. In parallel, TDP‐43 mislocalization to the cytoplasm was observed in ECs.

To further investigate the underlying mechanisms of BBB disruption, Cheemala et al. purified and cultured brain ECs from *Tardbp* ^*G348C/+*^ mice and observed increased permeability along with reduced levels of the cell junction proteins, including vascular endothelial (VE)‐cadherin, claudin‐5, and ZO‐1 relative to littermate controls. Cytoskeletal abnormalities were also evident, with alterations in actin filaments, microtubules, and focal adhesions, suggesting impaired structural integrity of the endothelium.

**FIGURE 2:**
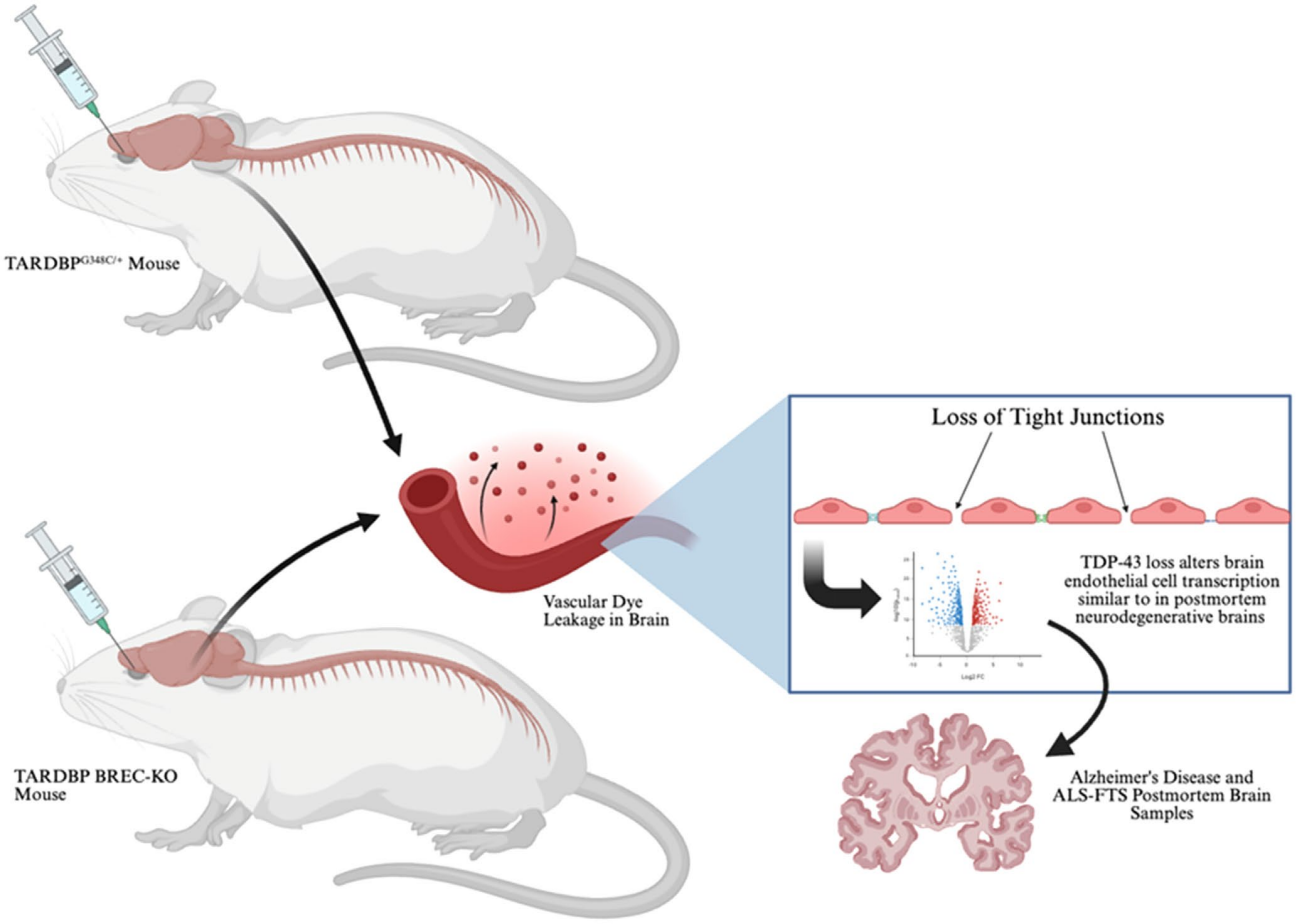
Loss of endothelial nuclear TDP‐43 drives blood–brain barrier (BBB) permeability and recapitulates transcriptomic signatures seen in human neurodegeneration.

To determine whether endothelial TDP‐43 loss is sufficient to drive BBB dysfunction, the authors generated a brain EC–specific *Tardbp* knockout model (BrEC‐KO). These mice exhibited pronounced BBB leakage, loss of VE‐cadherin, disruption of the actin cytoskeleton, and increased markers of neurovascular injury, including fibrin deposition, astrocyte activation, and microglial proliferation. The phenotype was more severe than *Tardbp* ^*G348C/+*^ mice, supporting a dose‐dependent effect of TDP‐43 loss. In addition, BrEC‐KO mice developed cognitive and behavioral impairments consistent with FTD‐like features. Moreover, transcriptomics analysis revealed overlapping gene expression changes between ECs from BrEC‐KO mice and siTDP‐43‐treated human brain ECs, whereas alterations were less pronounced in *Tardbp* ^*G348C/+*^ mice. Notably, genes involved in BBB maintenance were significantly downregulated, particularly within the Wnt/β‐catenin signaling pathway. Furthermore, differentially expressed genes (DEGs) in BrEC‐KO overlapped with those identified in human AD and ALS‐FTD brain tissue. These findings are supported by independent work from the same group, in which Omar et al. [20] demonstrated that a subset of ECs in patients with AD and ALS–FTD exhibits reduced TDP‐43 expression and impaired Wnt/β‐catenin signaling (Figure 2).

Although the previous study was focused on the loss of nuclear TDP‐43, Zamudio et al. [23] demonstrated that neuronal overexpression and aggregation of TDP‐43 in aged wild‐type (WT) mice also led to increased immune activation, including elevated immunoglobulin G (IgG) levels, and infiltration of CD3 and CD4^+^ T cells. Concurrent activation of ECs and pericytes suggested compromise of the BBB integrity. Moreover, BBB permeability rendered the frontal cortex more susceptible to systemic inflammatory challenges: following lipopolysaccharide (LPS) administration. TDP‐43‐overexpression mice exhibited pronounced neutrophil infiltration, neuronal loss, reduced synaptosomal‐associated protein 25 (SNAP‐25) expression, and behavioral impairments. Collectively, these findings indicate that both nuclear depletion and pathological aggregation of TDP‐43 converge on shared vascular and neuroinflammatory pathways, highlighting that TDP‐43 dysregulation can affect BBB integrity.

In summary, nuclear TDP‐43 plays a critical role in maintaining BBB homeostasis. Its loss in ECs leads to age‐dependent BBB dysfunction mediated by alterations in cell junction proteins and cytoskeletal defects. More broadly, TDP‐43 dysregulation promotes vascular instability and neuroinflammation, supporting the therapeutic rationale for restoring TDP‐43 nuclear function or Wnt/β‐catenin signaling to preserve BBB integrity and neuronal viability.

Earlier ultrastructural work by Garbuzova‐Davis et al. [9], provided initial evidence of barrier disruption in the SOD1‐G93A mice. Using electron microscopy, they identified BBB and BSCB abnormalities, including edema, inflammatory infiltration, mitochondrial damage, astrocyte degeneration, and endothelial injury surrounding spinal motor neurons in SOD1‐G93A mice. In a follow‐up study by Garbuzova‐Davis et al. [9], the same group evaluated BSCS integrity at two disease timepoints: 13 weeks of age (early symptomatic phase), and 17–18 weeks of age (late‐stage disease). Using Evans Blue dye to assess vascular permeability, they observed leakage in both cervical and lumbar spinal cord regions at both time points, with more pronounced endothelial dysfunction and BBB breakdown at advanced stages.

**FIGURE 3:**
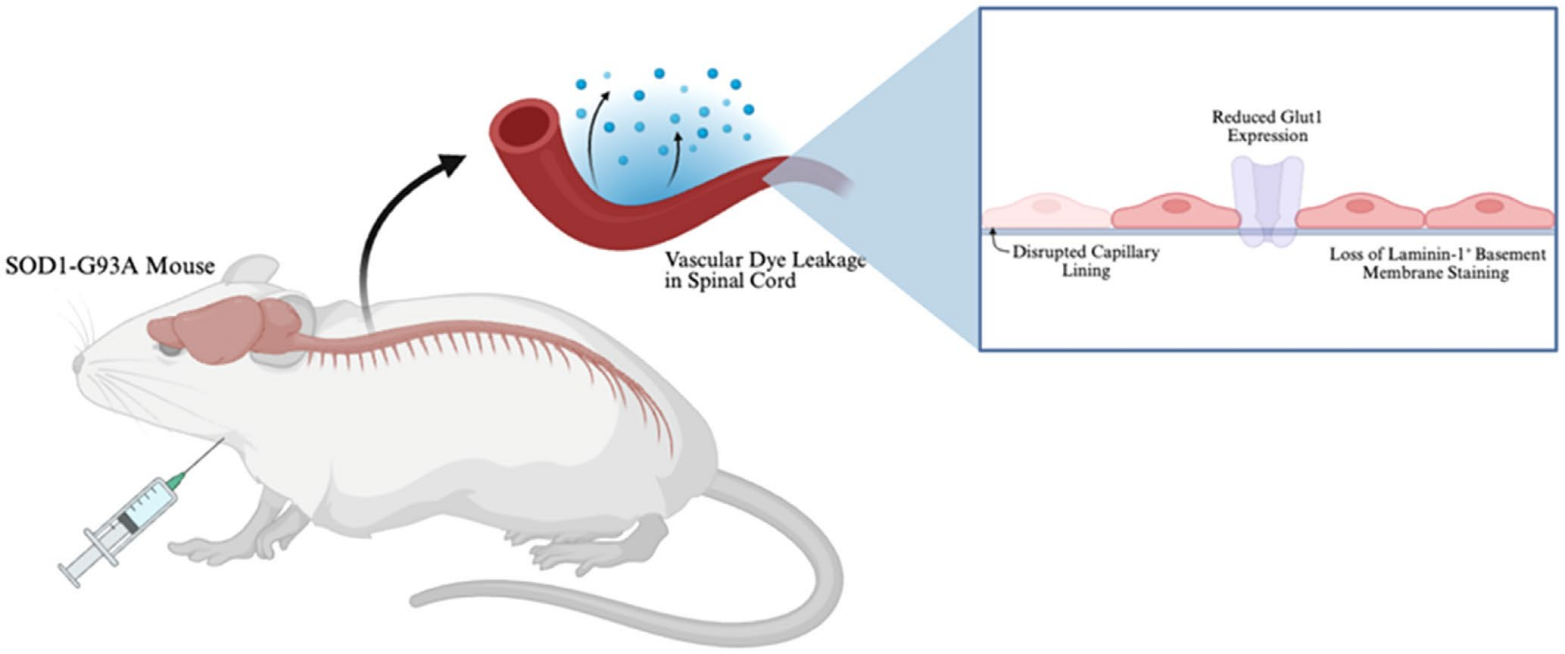
Early‐ and late‐stage SOD1‐G93A mice exhibited age‐dependent motor neuron degeneration and vascular disruption leading to Evans Blue leakage, beginning with early symptom onset and increasing with disease progression.

Immunohistochemical analysis further characterized the molecular and structural changes associated with BSCB disruption. Laminin‐1, a key basement membrane component, was significantly reduced in affected regions. Expression of Glut‐1, the principal endothelial glucose transporter in the CNS, was decreased and its localization disrupted, indicating impaired metabolic support. ECs exhibited morphological abnormalities, including swelling, degeneration, and intercellular gaps, consistent with disrupted CD146 staining. In parallel, marked astrogliosis was observed surrounding damaged vasculature and neural tissue, reinforcing the presence of a reactive neurovascular environment (Figure 3).

Based on these observations, the authors proposed that BSCB disruption permits the leakage of large plasma proteins, such as IgG, into the CNS. IgG accumulation may therefore serve as a surrogate marker of barrier dysfunction in ALS. Earlier studies by Engelhardt et al. [24] and Donnenfeld et al. [25] reported elevated IgG levels in the spinal cord and motor cortex tissues of postmortem patients with ALS. This is further supported by Zhong et al. [10], who demonstrated IgG deposition in the lumbar spinal cord across multiple SOD1 mouse models, and Nicaise [26]. Moreover, this work identified both IgG and hemosiderin in the brainstem and spinal cord of SOD1^G93A^ mice. Given its molecular size (approximately 150 kDa), IgG does not readily cross an intact BBB/BSCB. Hence, its presence within the CNS parenchyma is indicative of barrier leakage [27]. Similarly, hemosiderin, an iron‐storage protein complex derived from hemoglobin degradation, serves as a marker of vascular damage or microhemorrhage [28]. Taken together with earlier ultrastructural evidence, these findings support the concept that BSCB dysfunction is an early and progressive feature of ALS, closely associated with vascular disruption and immune activation. Such barrier compromise likely contributes to motor neuron vulnerability, establishing a mechanistic link between neurovascular dysfunction and neurodegeneration [8, 14].

Building on these observations, Zhong et al. [10] systematically investigated BSCB integrity in three mSOD1 mouse models (SOD1^G93A^, SOD1^G37R^, and SOD1^G85R^), each characterized by distinct disease kinetics. Animals were assessed at pre‐symptomatic and mid‐stage time points (SOD1^G93A^ mice at 2 and 3.5 months; SOD1^G37R^ mice at 3.5 and 5.5 months; SOD1^G85R^ mice at 6.5 and 11 months) [9]. Immunohistochemical analysis of lumbar spinal cord tissue revealed progressive IgG extravasation in all mutant lines, which was absent in wild‐type controls. The intensity of IgG deposition increased with disease progression, indicating worsening barrier permeability. In parallel, elevated hemosiderin levels were detected at early stages, suggesting that vascular injury precedes overt neurodegeneration. Further, immunostaining of lumbar spinal cords from these mice revealed IgG deposits in all three SOD1 mouse models, but not in SOD1^WT^ mice. The intensity of IgG immunostaining increased as the disease progressed in mSOD1 mice. The presence of IgG in the spinal cords indicates BSCB disruption, allowing highly regulated proteins to enter the lumbar spinal cord parenchyma. Hemosiderin, a marker of vascular leakage, was also elevated in early mSOD1 mice compared to WT‐SOD1, suggesting that early vascular injury contributes to BSCB disruption. Most significantly, Zhong et al. found that this breakdown begins before motor neuron degeneration and symptom onset, suggesting that BSCB breakdown may contribute to motor neuron degeneration rather than merely be a consequence. As barrier breakdown triggers an immune response, Zhong et al. [10] examined markers of barrier damage, inflammation, and immune‐activated endothelium to determine when immune changes occur during disease progression. They found that these markers were not expressed following BSCB damage, indicating that the neurovascular inflammatory response occurs later in disease progression. To further characterize the structural basis of barrier failure, protein expression analyses demonstrated early reductions in tight junction components, including ZO‐1, occludin, and claudin‐5, in the spinal cords of mSOD1 mice. Morphologically, the anterior horn, rich in motor neurons, exhibited reduced capillary density and extracellular edema. In SOD1^G93A^ mice, spinal cord blood flow was reduced by approximately 30%–45% at early stages, particularly within the anterior horn. This regional hypoperfusion may impair oxygen delivery, thereby contributing to selective motor neuron degeneration.

The importance of vascular integrity for motor neurons' survival is further supported by studies on vascular endothelial growth factor (VEGF). Oosthuyse et al. [29] demonstrated that impaired VEGF signaling induces motor neuron degeneration under hypoxic conditions, even in non‐SOD1 models. Similarly, Lambrechts et al. [30] showed that reduced VEGF expression exacerbated disease severity and decreased survival in SOD1^G93A^ mice, highlighting the detrimental impact of impaired angiogenesis and tissue hypoxia.

**FIGURE 4:**
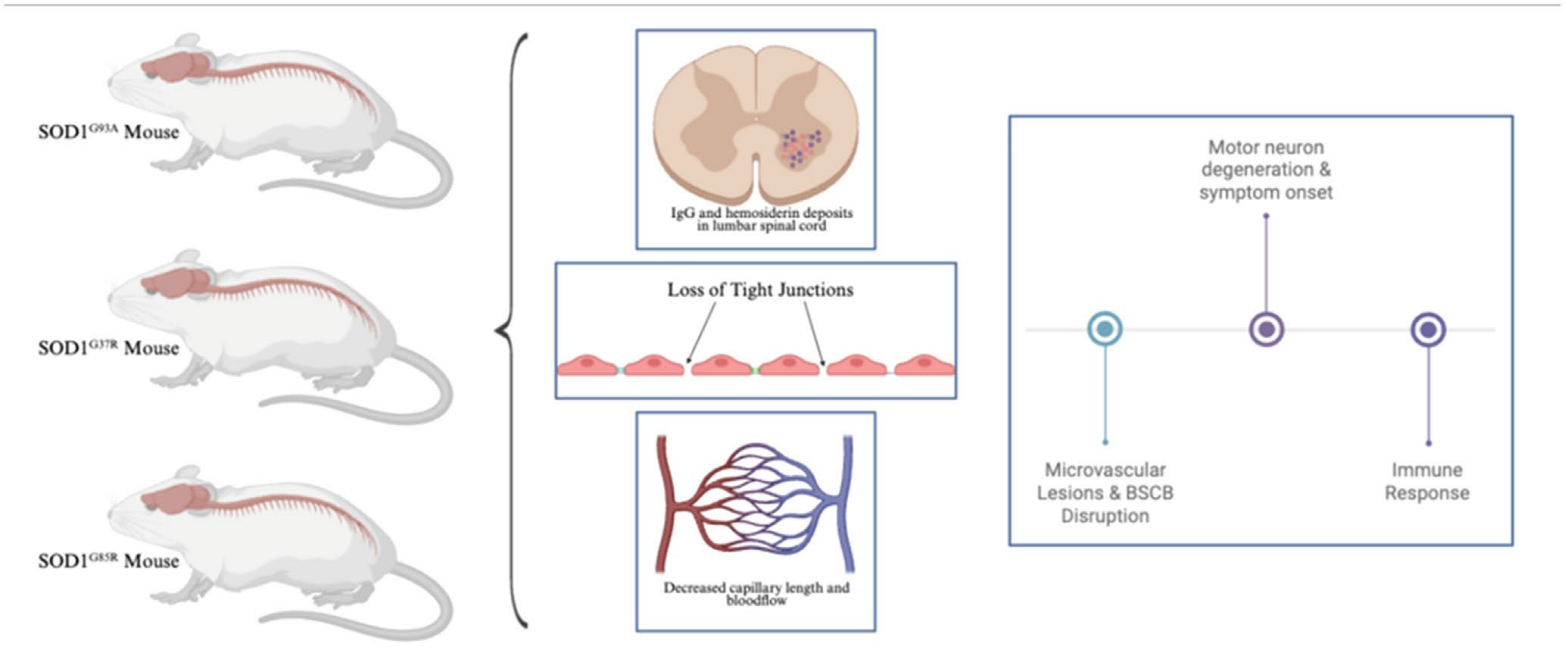
Mutant SOD1 (mSOD1) ALS mouse models, SOD1^G93A^, SOD1^G37R^, and SOD1^G85R^ (top, middle, and bottom, respectively) display pre‐symptomatic and pre‐motor neuron degeneration alterations in blood–brain barrier function.

Collectively, these studies position BSCB breakdown and reduced spinal cord perfusion as early, upstream events in ALS pathophysiology. Therapeutic strategies aimed at preserving barrier integrity and restoring vascular function may therefore represent a rational approach to slowing or potentially modifying the neurodegenerative cascade [31, 32, 33] (Figure 4).

Winkler et al. [11] investigated the temporal relationship between BBB and BSCB disruption and motor neuron degeneration in SOD1^G93A^ mice and evaluated therapeutic strategies targeting barrier dysfunction.

SOD1^G93A^ and SOD1^WT^ mice were treated with low‐dose warfarin, an anticoagulant, or saline to modulate microvascular integrity. Warfarin treatment induced a dose‐dependent increase in microvascular damage, evidenced by accumulation of IgG, hemoglobin, hemosiderin, and free iron within the lumbar anterior horn. Notably, approximately 85% of vascular lesions are localized to spinal cord capillaries. Disease onset was accelerated in a dose‐dependent manner in warfarin‐treated SOD1G93A mice, supporting a pathogenic role of vascular injury. The presence of free iron is particularly relevant, as it catalyzes the formation of reactive oxygen species (ROS), thereby amplifying oxidative stress and contributing to neurodegeneration [34, 35]. Consistent with this, increased oxidative damage correlated with iron accumulation. Warfarin‐treated mSOD1 mice also exhibited reduced neurite density, loss of choline acetyltransferase (ChAT)‐positive motor neurons, and increased ubiquitin‐positive aggregates. Importantly, microglial and astrocytic activation were not observed at presymptomatic stages, reinforcing earlier findings by Zhong et al. [10] that barrier disruption precedes overt neuroinflammation. In vitro, warfarin had no direct effect on endothelial permeability or TJ protein expression, indicating that the observed vascular pathology reflects intrinsic barrier vulnerability rather than a direct pharmacological effect. Furthermore, three potential therapeutics were evaluated, each aimed at addressing either a temporal injury to the BBB or downstream consequences of BBB disruption. An activated protein C analog (5A‐APC), known to protect against BBB damage in CNS injury, preserved BSCB integrity in warfarin‐treated SOD1^G93A^ mice. Barrier permeability was normalized, as evidenced by the absence of leakage into the CNS. Motor impairment and neurodegeneration were delayed compared to untreated warfarin‐ and saline‐treated SOD1^G93A^ mice, and oxidative damage was reduced. As SOD1 protein and mRNA levels remained unchanged, 5A‐APC appears to act by restoring BBB integrity and preventing the entry of proteins and free iron into the CNS, rather than by modulating SOD1 expression. Moreover, 5A‐APC has been shown to exert anti‐inflammatory effects, although pre‐symptomatic treatment did not alter the microglial response. Deferasirox (DFX), an iron chelator, did not alter barrier permeability but reduced free‐iron accumulation and delayed disease onset and motor neuron degeneration. Oxidative stress associated with the SOD1 mutation was also reduced. Glutathione monoethyl ester (GSHE) is a potent, lipophilic derivative of glutathione (GSH) that functions as an antioxidant and, importantly, as a prodrug to replenish intracellular glutathione levels [36]. GSHE is cell‐permeable and can cross the BSCB, whereas GSH is poorly transported into cells. GSHE was used as an antioxidant to mitigate damage induced by ROS and SOD1 protein aggregation. Similar to DFX, GSHE did not improve barrier integrity or iron accumulation in SOD1^G93A^ mice, but it delayed motor symptom onset and motor neuron degeneration, with a concomitant reduction in oxidative stress. In addition, the authors employed a mutant form of activated protein C (APC) mutant, 5A‐APC, which retains cytoprotective signaling properties but lacks more than 90% of its anticoagulant activity, to assess whether BSCB repair could delay motor neuron injury in SOD1^G93A^ mice. Treatment with 5A‐APC prevented early motor‐neuron degeneration, as evidenced by normalization of neuritic density and clearance of ubiquitin‐positive aggregates associated with both spontaneous and accelerated BSCB breakdown. Notably, BSCB protection was the primary beneficial effect during the early stage of the disease in SOD1^G93A^ mice.

Among the tested interventions, 5A‐APC proved to be the most effective therapy, extending both lifespan and the disease progression phase compared to warfarin‐ and saline‐treated mSOD1 mice. These effects were observed in both spontaneous and experimentally induced microvascular lesions. The results underscore the importance of initiating 5A‐APC treatment at a pre‐symptomatic stage rather than after symptom onset. This benefit is likely mediated by the restoration and/or maintenance of BSCB integrity, thereby preventing the entry and accumulation of neurotoxic blood‐derived substances in the spinal cord during the early stages of disease.

**FIGURE 5:**
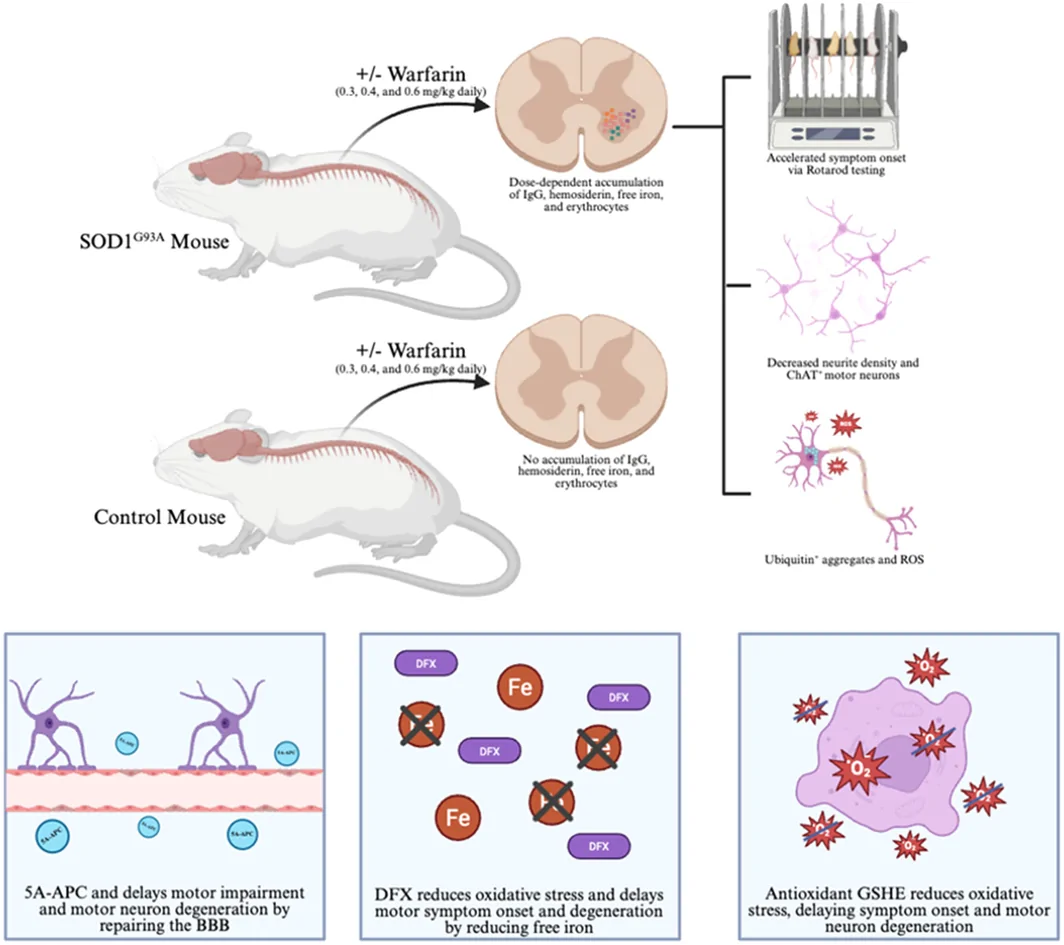
Blood–spinal cord barrier (BSCB) disruption accelerates motor neuron degeneration in ALS models and highlights therapeutic rescue strategies. In SOD1^G93A^ mice, low‐dose warfarin administration induces dose‐dependent BSCB breakdown, leading to spinal cord accumulation of IgG, hemosiderin, free iron, and erythrocyte pathological features absent in warfarin‐treated control mice. Barrier disruption is associated with accelerated symptom onset, reduced neurite density, loss of ChAT^+^ motor neurons, increased ubiquitin aggregates, and ROS. Therapeutic interventions targeting distinct downstream mechanisms demonstrate disease‐modifying effects: 5A‐APC preserves barrier integrity and delays motor impairment; DFX mitigates iron‐mediated oxidative stress; and GSHE reduces ROS burden, collectively delaying symptom onset and motor neuron degeneration.

Overall, the data [11] support BBB dysfunction as a key driver of neurodegeneration in mSOD1 mice. Exacerbation of vascular damage through warfarin treatment accelerated disease onset compared to saline‐treated mSOD1 mice, and these findings are consistent with Zhong et al. [10], demonstrating that the immune response follows barrier disruption and symptom onset. Moreover, this study highlights the role of BSCB in clinical disease progression and its role as a therapeutic target. BSCB breakdown contributes to the pathogenesis of early‐stage disease, and restoration of BSCB integrity can slow disease progression. These findings are relevant to patients with ALS, in which vascular pathology is increasingly recognized as a contributing factor (all REFs). Additionally, therapies targeting various BBB‐related mechanisms of neurodegeneration have been shown to extend lifespan in preclinical models. However, the precise role of BSCB dysfunction in the clinical progression of motor neuron degeneration remains to be fully elucidated. Further studies are required to determine whether early restoration of BSCB integrity can effectively slow disease progression in both preclinical models and patients with ALS (Figure 5).

Pan et al. [15] were the first to utilize a transgenic *C9orf72* BAC (C9‐BAC) mouse model expressing a hexanucleotide repeat expansion in the human *C9orf72* gene, the most common genetic cause of ALS. They aimed to characterize BBB dysfunction previously observed in other humanized mouse models of ALS. Following an in situ transcardial brain perfusion, BBB permeability, P‐gp, fatty acid, amino acid transport, and GLUT1 function were assessed at an early symptomatic stage. These processes have all been implicated in BBB stability and are known to be altered in ALS models.

Microvascular volume, assessed using ^14^C‐sucrose, did not differ between WT and C9‐BAC mice, with no significant differences between groups. Similarly, transport of ^3^H‐diazepam and ^14^C‐caffeine was unchanged, indicating preserved passive permeability of the BBB in C9‐BAC mice. Transport of ^3^H‐oleic acid, ^3^H‐digoxin, and ^14^C‐(L)‐alanine transport was also unaltered, suggesting that P‐gp, fatty acid, and amino acid transport systems remain intact. In contrast, glucose transport was increased in C9‐BAC mice: flux of 3H‐2‐deoxy‐D‐glucose (^3^H‐2DG) across the BBB was approximately 1.4 times higher compared to WT control mice, accompanied by a 1.3‐fold increase in brain microvascular GLUT1 expression. Other BBB functions, including passive diffusion and efflux transporter activity, remained unaffected.

Because BBB glucose entry is largely mediated by GLUT1, the increased BBB transport of ^3^H‐2DG likely reflects a compensatory mechanism to maintain cerebral glucose supply in the context of reduced neuronal uptake, as suggested by positron emission tomography (PET) imaging studies. Consistently, GLUT1 expression was elevated in brain microvessel‐enriched fractions (MEFs) from C9‐BAC mice, aligning with increased BBB glucose transport. Elucidating the upstream drivers of GLUT1 upregulation and enhanced transport capacity may provide a viable strategy for CNS drug delivery in ALS, including glucose‐mimetic conjugation approaches that exploit GLUT1‐mediated BBB transport [12, 18, 20, 37].

Proteomic analysis was conducted on mouse brain ECs, excluding cells of the neurovascular unit such as pericytes, astrocytes, and neurons. The TJ protein ZO‐1 was significantly reduced (14.3%) in C9‐BAC ECs. This finding is consistent with studies in mSOD1 and TDP‐43 mouse models, which have reported that reductions in ZO‐1 are associated with impaired BBB function [10, 22]. In addition, several endogenous transport proteins were differentially expressed between WT and C9‐BAC mouse ECs.

Overall, Pan et al. [15] did not detect vascular leakage or increased BBB permeability in the C9‐BAC mouse model at an early symptomatic disease stage. The authors note that the spinal cord vasculature was not assessed, despite the fact that BSCB dysfunction is frequently observed in mSOD1 mice. The absence of BBB disruption contrasts with findings from other ALS models, including mSOD1 and TDP‐43 mice, in which BBB dysregulation has been reported across multiple stages of disease progression, including the presymptomatic phase [9, 10, 11, 22]. Furthermore, the observed upregulation of GLUT1 differs from results in other mouse models, although the authors suggest that methodological differences may account for this discrepancy.

**FIGURE 6:**
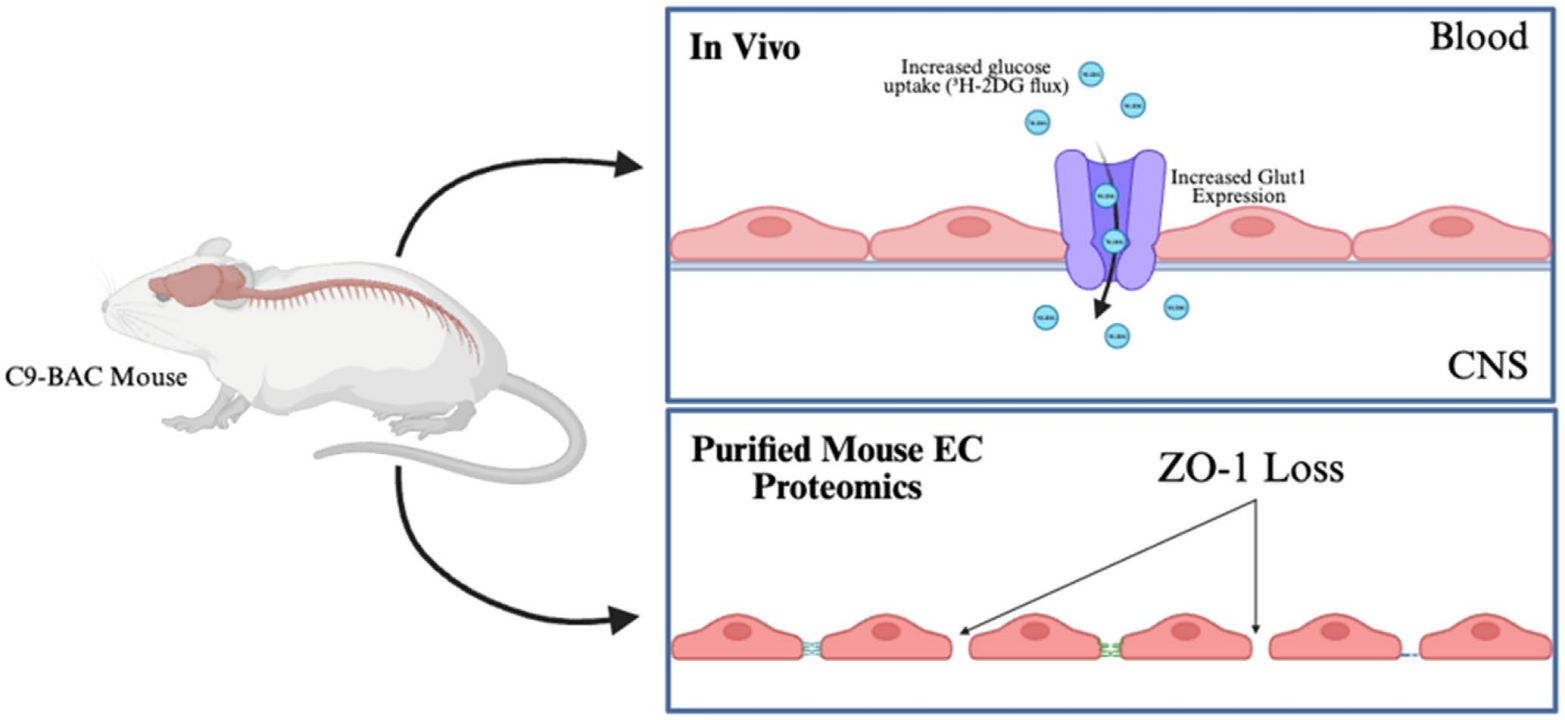
Human *C9orf72* BAC hexanucleotide expansion mice showed alterations in blood–brain barrier transport, though no vascular leakiness.

Compared with other *C9orf72* models and samples, brain ECs derived from patients with *C9orf72*‐associated ALS exhibited disruption of the EC layer, particularly when co‐cultured with C9‐BAC astrocytes [38]. This model also showed increased GLUT1 expression, supporting the findings of Pan et al. [15] (Figure 6).

Although early evidence of BBB/BSCB barrier dysfunction was largely established in preclinical SOD1 ALS models, we agree that the broader clinical relevance of these findings must be considered within the context of non‐SOD1 ALS models. Recent studies in non‐SOD1 ALS models reported in this manuscript, including TDP‐43 and *C9orf72*, collectively demonstrate that neurovascular dysfunction is not restricted to mutant SOD1 pathology. In particular, emerging evidence indicates that endothelial TDP‐43 loss disrupts Wnt/β‐catenin signaling, TJs integrity, and BBB homeostasis, while *C9orf72*‐associated models exhibit alterations in endothelial transport and barrier‐associated proteins. These findings suggest that vascular dysfunction may represent a convergent pathogenic mechanism across multiple ALS subtypes, including sporadic ALS, which is characterized by TDP‐43 pathology, thereby enhancing the translational and clinical relevance of the review [38, 39].

## 5 Novel Strategies to Deliver Drugs Across the Blood–Brain Barrier

### 5.1 Ultrasound‐Assisted Drug Delivery Techniques Across the BBB in ALS

Despite advances in understanding ALS biology, therapeutic development remains challenging due to limited drug penetration into the CNS, where motor neuron degeneration primarily occurs [3]. The modest efficacy of current treatments for ALS, such as EDR and RZ, may partially reflect the restrictive properties of the BBB and BSCB, which limit drug delivery to the CNS. Given these challenges, a comprehensive evaluation of novel strategies for non‐invasive, localized, and targeted drug delivery to the motor cortex or spinal cord is essential. Approaches aimed at bypassing or transiently modulating the BBB may enhance therapeutic efficacy and improve clinical outcomes in ALS [12, 18]. One such strategy is focused ultrasound (FUS), which operates through acoustic cavitation of intravenously administered microbubbles (MBs) at targeted sites. This process induces transient and reversible modulation of endothelial TJs without causing significant tissue damage, thereby enhancing regional drug delivery within the CNS.

#### 5.1.1 Mechanisms of Acoustically Mediated BBB Opening

Microbubble (MB)‐assisted focused ultrasound (FUS) transiently permeabilizes the BBB by combining low‐intensity ultrasound with intravenously injected gas‐core MBs. Under the applied acoustic field, MBs undergo stable oscillation (and, at higher pressures, inertial cavitation) within cerebral capillaries, generating localized mechanical stresses, microstreaming, shear stress, and transient pore formation that loosen endothelial TJs and increase transcytosis, producing a reversible, spatially confined increase in BBB permeability without the thermal ablation associated with high‐intensity FUS. This approach enhances drug extravasation and bioavailability across the BBB in a safe, non‐invasive, and spatially localized manner [40]. More recently, perfluorocarbon nanodroplets (NDs) have emerged as candidates to replace conventional microbubbles [40]. These smaller, more stable liquid‐core precursors vaporize into microbubbles in situ upon ultrasound exposure, offering longer circulation time, extravasation into the perivascular space prior to activation, and finer control over the timing and location of BBB opening.

**FIGURE 7:**
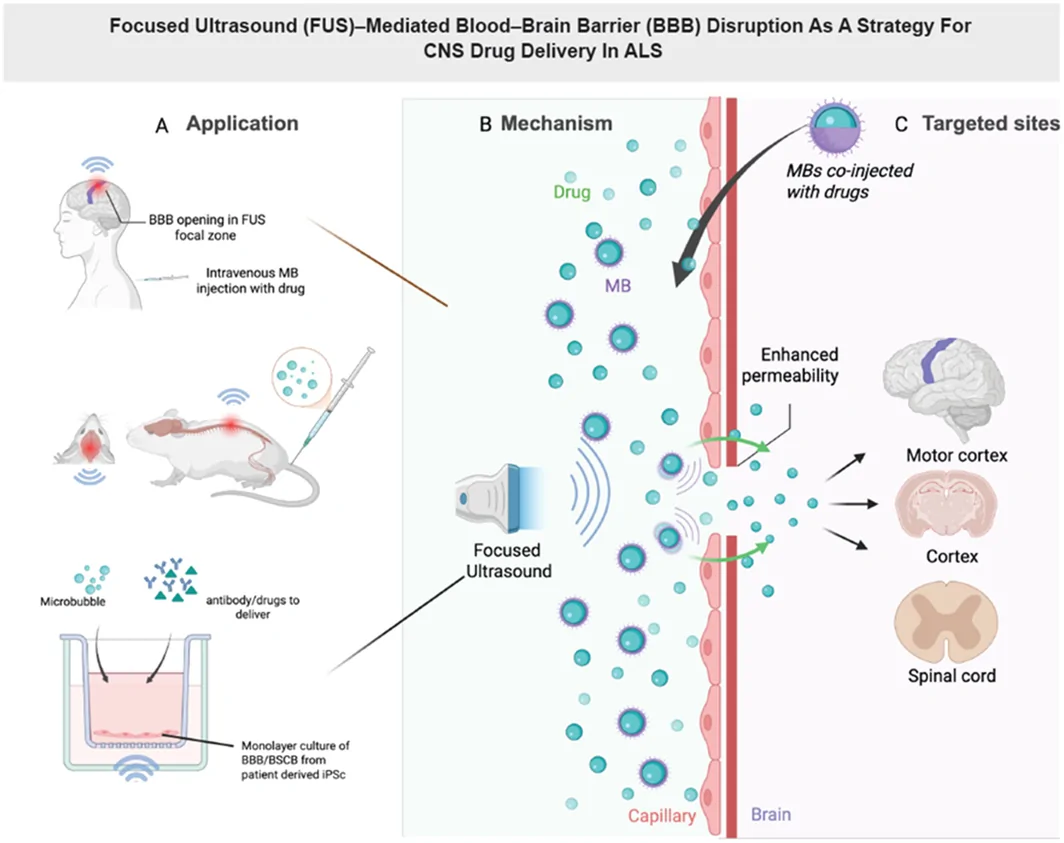
Schematic illustrating focused ultrasound (FUS)–mediated blood–brain barrier (BBB) disruption as a strategy for CNS drug delivery in ALS. (A) FUS combined with microbubbles (MBs) has been explored in preclinical ALS models and is being evaluated in clinical settings. Approaches include transcranial FUS in patients targeting the motor cortex, intravenous drug delivery combined with FUS in rodents targeting the BBB and BSCB, and an in vitro platform derived from patient iPSCs. (B) Mechanism: Intravenously administered MBs oscillate under FUS exposure, producing mechanical effects that transiently open the BBB and enhance drug permeability. Drugs can be co‐injected with MBs or conjugated to them directly, allowing localized and noninvasive delivery to the CNS. (C) Targeted sites: FUS enables spatially selective targeting of ALS‐relevant regions such as the motor cortex, cerebral cortex, and spinal cord, thereby enhancing therapeutic precision while minimizing systemic side effects.

To enhance drug penetration into the CNS, MBs are administered intravenously and subsequently activated by FUS at the targeted site, without causing damage to surrounding tissues. The oscillation of these MBs induces a transient and reversible opening of TJs between ECs of the BBB and BSCB. This controlled modulation allows therapeutic agents, including antibodies, growth factors, and ASOs, to enter targeted brain regions at higher concentrations than would otherwise be achievable [37, 41, 42, 43] (Figure 7).

#### 5.1.2 Safety Considerations

In the first‐in‐human ALS trial, gadolinium leakage confirmed successful BBB opening (approximately 350 mm^3^ of motor cortex volume, delivered as two 175 mm^3^ spots) at the sonicated motor cortex in all four subjects, which normalized within 24 h, with no serious clinical, radiologic, or electroencephalographic adverse events [18].

This demonstrated that non‐invasive permeabilization over eloquent cortex is feasible and reversible even in a neurodegenerative disease population. Residual risks: petechial microhemorrhage, edema, and off‐target sonication at skull interfaces are managed through MRI guidance, patient‐specific skull density correction, and conservative pressure margins below the inertial cavitation threshold. Neurological status in this trial was tracked using standardized MRC manual muscle strength grading across upper and lower extremity muscle groups bilaterally (normal sum scores: 35/arm, 30/leg), alongside safety laboratory panels (complete blood count, inflammatory markers, renal function, liver enzymes, electrolytes) and multi‐sequence safety MRI (T1 ± gadolinium, T2, FLAIR, GRE, DWI) to detect subclinical hemorrhage, edema, or ischemia. Although this study confirmed the technical feasibility and tolerability of FUS in patients with ALS, it did not assess therapeutic efficacy, highlighting the need for further translational research [16]. To build on these initial clinical findings and evaluate the therapeutic potential of FUS in combination with FDA‐approved agents, several recent in vivo and in vitro studies have been conducted in ALS models.

#### 5.1.3 Preclinical and Clinical Developments in Neurodegenerative Disease

Acoustically mediated BBB opening represents a rapidly advancing strategy across neurodegenerative diseases more broadly, with the potential both to improve delivery of already‐approved therapeutics and to enable entirely new classes of CNS‐directed molecules that are currently BBB‐limited. Within this landscape, ExAblate Neuro has generated the majority of current clinical evidence, across ALS, Parkinson's disease, and Alzheimer's disease. Although device platforms with primarily oncology‐focused trial histories (NaviFUS, SonoCloud) and applications in Huntington's disease remain earlier‐stage, proposed extensions of the same underlying approach. Building on this broader context, the following section focuses specifically on preclinical and clinical evidence in ALS [1].

A recent pilot study in preclinical a SOD1^G93A^ ALS mouse model demonstrated enhanced delivery of EDR to the motor cortex using FUS, with no evidence of gross tissue damage [16]. This approach was associated with improved motor function, reduced levels of misfolded SOD1, and attenuation of pathological markers, including astrocytosis and microgliosis. Notably, the study showed significantly greater CNS penetration compared to systemic delivery alone, underscoring the capacity of FUS to facilitate the delivery of therapeutics that are otherwise BBB‐impermeable [16]. A complementary study employing LIPUS reported delayed disease onset and prolonged survival in SOD1^G93A^ mice [44], likely mediated by increased cerebral blood flow in the motor cortex, improved endothelial integrity, enhanced microvascular density, and upregulation of neurotrophic factors, potentially via activation of the transient receptor potential vanilloid 4 (Trpv4) ion channel. Although therapeutic efficacy was not the primary endpoint, this study supports the biological plausibility and technical feasibility of ultrasound‐mediated BBB modulation in ALS and provides a foundation for future applications in targeted drug delivery and gene therapy [45].

Expanding the anatomical scope, Montero and colleagues [45] investigated repeated FUS targeting the lumbar spinal cord in SOD1^G93A^ mice to induce transient barrier disruption at the level of the BSCB, in combination with co‐administration of insulin‐like growth factor 1 (IGF1) [45]. Importantly, the study also demonstrated that FUS alone could extend survival in this ALS model, potentially through modulation of reactive microglial cells and increased infiltration of neuroprotective, anti‐inflammatory T lymphocytes into the spinal cord.

Although ultrasound‐mediated BBB modulation has been successfully demonstrated in patients with ALS, the molecular effects of FUS + MBs on human ALS BBB cells remain poorly understood, which may limit their therapeutic translation. To address this gap, researchers have recently employed induced pluripotent stem cell (iPSC)‐derived brain endothelial‐like cells from patients with ALS, including carriers of the *C9orf72* repeat expansion, to investigate the effects of FUS + MB on human BBB models. This in vitro study is the first to demonstrate enhanced transport of large molecules, such as TDP‐43‐targeting antibodies, across an ALS BBB model. In addition, it provides initial mechanistic insights into how FUS + MB may facilitate therapeutic antibody delivery in human‐derived systems [17].

### 5.2 Limitations

Although these key translational advances position FUS as a promising non‐invasive platform for targeted CNS drug delivery in ALS, several important limitations remain. There is a need to evaluate combination strategies incorporating FUS with a broader range of ALS therapeutics beyond EDR and IGF‐1. Expanding the number of animal studies, clinical trials, and patient‐derived iPSC models will be essential to improve reproducibility and translatability. Optimizing sonication parameters is critical to minimize potential adverse effects, including microhemorrhage, inflammation, and off‐target neuronal injury, and the development of personalized FUS protocols based on patient‐specific neuroimaging may further enhance targeting precision and safety by accounting for anatomical and disease‐related variability. Future approaches should consider simultaneous targeting of both the motor cortex and spinal cord to improve drug distribution and overall therapeutic efficacy. Such strategies may be particularly relevant for the delivery of antibodies, ASOs, neurotrophic factors, and gene therapies, where broader CNS coverage could offer advantages over localized treatment.

More fundamentally, no trial to date has been powered to detect a functional or disease‐modifying clinical benefit; BBB opening itself has been reliably demonstrated, but therapeutic efficacy following it has not. Preclinical‐to‐clinical translation is also complicated by interspecies differences in skull thickness and acoustic attenuation, requiring substantial parameter re‐calibration from rodent models, and by the practical reality that MRI‐guided platforms remain confined to specialized centers with standardized protocols not yet harmonized across the different device types in clinical use.

### 5.3 Viral Vector Mediated Delivery in Preclinical Models of ALS

The investigations carried out by Wang et al. [46] evaluated systemic administration of scAAV9‐IGF1 in humanized SOD1^G93A^ transgenic ALS mice to assess BBB permeability, neuromotor function, and reduction of neurofilament pathology in the CNS and spinal cord. Systemic delivery of scAAV9‐IGF1 resulted in robust downregulation of *SOD1* expression and was associated with prolonged survival, delayed motor impairment, and reduced axonal damage. Mechanistically, scAAV9‐IGF1 inhibited the activation of p38 MAPK and JNK‐mediated apoptotic pathway by reducing phosphorylation of p38 and JNK. This was accompanied by upregulation of the anti‐apoptotic protein Bcl2 and suppression of pro‐apoptotic factors, including BAX, caspase‐9, and caspase‐3 [46].

TDP‐43 is a vital nuclear protein whose aggregation and mislocalization contribute to ALS pathology, including cytoplasmic stress granules, neuronal loss, and degeneration. Amado et al. [47] investigated a gene‐silencing approach using ASO and AAV9‐mediated RNA interference targeting *Atxn2*, a stress granule–associated protein, in TAR4/4 transgenic mice, a model of sporadic ALS. Intrathecal administration of miAtxn2 resulted in downregulation of TDP‐43 expression and attenuation of TDP‐43‐mediated toxicity. This was associated with improved motor strength and survival, enhanced neuromotor function, reduced neuroinflammation, and preservation of neuronal viability [48].

The pathogenic role of TDP‐43 proteinopathy is further supported by Mallika et al. [49], who demonstrated that intravenous administration of a BBB‐permeable AAV capsid expressing CTR (AAV‐PHP.eB‐CTR) in *ChAT‐Cre; Tardbp* ^*f/f*^ transgenic mice significantly restored neuromotor function, prevented paralysis, and slowed the disease progression.

Recently, Keeler et al. [50] investigated an adeno‐associated virus (AAV) mediated gene‐silencing strategy using a microRNA (miRNA) targeting SOD1 (AAV‐miRSOD1) to improve respiratory function and survival in SOD1^G93A^ mice. The vector was delivered through intralingual and intrapleural injections, with respiratory and behavioral parameters monitored until the end stage. Effective SOD1 silencing was confirmed in the diaphragm, tongue, and CNS. This intervention extended survival by approximately 50 days and delayed both weight loss and limb weakness compared to controls. Histological analysis demonstrated preservation of neuromuscular junctions and axonal integrity in phrenic and hypoglossal nerves. Although SOD1 suppression improved respiratory function and overall survival, it did not reverse the restrictive lung phenotype. These findings indicate that targeted silencing of SOD in respiratory motor neurons can significantly prolong survival and sustain respiratory function in SOD1^G93A^ ALS mice. Importantly, the study underscores the therapeutic relevance of specifically targeting respiratory motor units in ALS. However, an optimal treatment strategy would likely require broader CNS coverage to address motor units involved in both respiration and limb function.

Interpreting AAV gene‐therapy studies across ALS literature remains inherently challenging. Reported outcomes vary substantially due to differences in injection routes, capsid serotypes, vector designs, titration methods, dosing paradigms, and the age at treatment. This methodological heterogeneity limits direct comparability between studies and complicates the establishment of a clear consensus on therapeutic efficacy. Notably, although multiple investigations have assessed alternative delivery routes in SOD1 mouse models, results have been inconsistent, highlighting that both vector biology and route of administration critically influence transduction efficiency and therapeutic response.

### 5.4 Challenges Associated With AAV‐Based Delivery Platforms

AAVs are among the most widely used viral vector platforms for delivering genetic material to the CNS, particularly in ALS [51]. Their low immunogenicity, non‐pathogenic nature, and capacity for long‐term transgene expression make them attractive therapeutic candidates. However, efficient CNS delivery remains limited by the BBB, which restricts the entry of large molecules, including viral vectors [52].

Although certain serotypes, such as AAV9 and AAVrh10, can partially cross the BBB following systemic administration, a substantial proportion of the viral particles remain sequestered in peripheral organs, particularly the liver and spleen [53]. As a result, CNS transduction efficiency is reduced, often necessitating high vector doses to achieve therapeutic effects. These high doses are associated with off‐target toxicity, undesirable immune responses, and other adverse effects [51]. Systemic AAV administration has been linked to toxicities in multiple organs, including the heart, liver, lungs, and kidneys, and is further complicated by both innate and adaptive immune responses, which can also neutralize the vector [54]. At the molecular level, innate immune activation is mediated by pattern recognition receptors such as MDA5 and RIG‐I, as well as Toll‐like receptor (TLR) 9, which detects unmethylated CpG motifs within the AAV genome and capsid. This triggers downstream activation of inflammatory pathways, including TLR2 signaling, leading to cytokine production and potential tissue injury. Antigen‐presenting cells, such as macrophages and dendritic cells, further amplify the immune response by activating B and T lymphocytes, resulting in the generation of neutralizing antibodies against AAV capsids [55]. Additional limitations include suboptimal cellular uptake, restricted packaging capacity (which limits delivery of large or dual genetic constructs), and variability in transduction efficiency across tissues [56]. Beyond these biological constraints, practical challenges such as vector purity, stability, large‐scale manufacturing, and clinical translatability continue to impede the widespread therapeutic application of AAV‐based approaches [57, 58].

### 5.5 Approaches for Optimizing the Viral Vector Delivery for ALS

Different viral vectors have been explored for therapeutic delivery in ALS, including adenoviruses, retroviruses, herpes simplex viruses, AAV, and lentivirus vectors [59]. It is now established that ALS is a heterogeneous disease, with factors such as genetic background, age, sex, clinical phenotype, and ethnicity influencing disease onset and progression [60]. To address this heterogeneity, optimization of viral vector systems, particularly AAVs, has been proposed to improve therapeutic efficacy. Key objectives include enhancing BBB permeability, increasing transduction efficiency, and minimizing immunogenicity, thereby improving CNS targeting and clinical outcomes. Several strategies have been developed to optimize AAV vectors, including those listed below:

#### 5.5.1 Capsid Engineering

AAV's features, or capsid profiles, encompass BBB penetration ability, immunogenicity, cellular tropism, and tissue tropism. Amino acid sequences of the cap gene encoding the viral capsid proteins determine the capsid profile, and modulation of these sequences allows for the maneuvering of biological properties. Given that the BBB represents a major barrier to CNS delivery, engineered AAV variants with enhanced brain tropism have been developed to improve transduction efficiency and facilitate BBB penetration [61]. In genetically modified AAVs, highly immunogenic epitopes and neutralizing antibody binding sites can be reduced or eliminated, resulting in improved neural and endothelial targeting with lower immunogenicity [62]. A landmark study conducted by Deverman et al. [63] introduced the engineered capsid‐AAV PHP.B, which demonstrated a 50–100‐fold increase in CNS transduction efficiency. This was achieved using a Cre recombination–based directed evolution approach, in which large libraries of capsid variants were generated by inserting randomized peptide sequences into variable regions of the viral capsid, followed by in vivo selection for enhanced CNS tropism.

#### 5.5.2 Enhancing Promoter Specificity

A critical determinant of AAV‐mediated gene delivery is transgene expression, which is governed by the choice of promoter and directly influences cell‐type specificity. In gene therapy applications, particularly those targeting the CNS, promoters are selected to drive high and selective expression in specific cell populations. Commonly used promoters include human synapsin‐1 (hSyn1) for neuron‐specific expression, glial fibrillary acidic protein (GFAP) for astrocyte‐restricted expression, and cytomegalovirus (CMV) for broad, ubiquitous expression. The combination of neuron‐specific promoters such as hSyn1 with engineered capsids like AAV‐PHP.B has been shown to further enhance targeting efficiency and specificity within the CNS [64].

#### 5.5.3 Persistence

The efficacy of AAV‐mediated gene therapy is closely linked to the persistence of transgene expression. However, repeated administration of AAVs carries a substantial risk of immunogenic responses. Following initial exposure, the host immune system generates both humoral and cellular immunity, including B‐ and T‐cell activation. Upon re‐administration, these responses are amplified, leading to neutralizing antibody formation and immune‐mediated cytotoxicity against transduced cells [65]. In contrast, repeated administration of ASOs is associated with a comparatively low risk of immunogenicity [66]. Sustained transgene expression and persistence can be improved by overcoming several key mechanisms of loss: (i) immune‐mediated clearance of transduced cells or suppression of transgene expression; (ii) dilution or loss of episomal AAV genomes due to cellular turnover in the absence of genomic integration; and (iii) epigenetic silencing of the transgene [61].

#### 5.5.4 Immunomodulation and CNS Transduction

**FIGURE 8:**
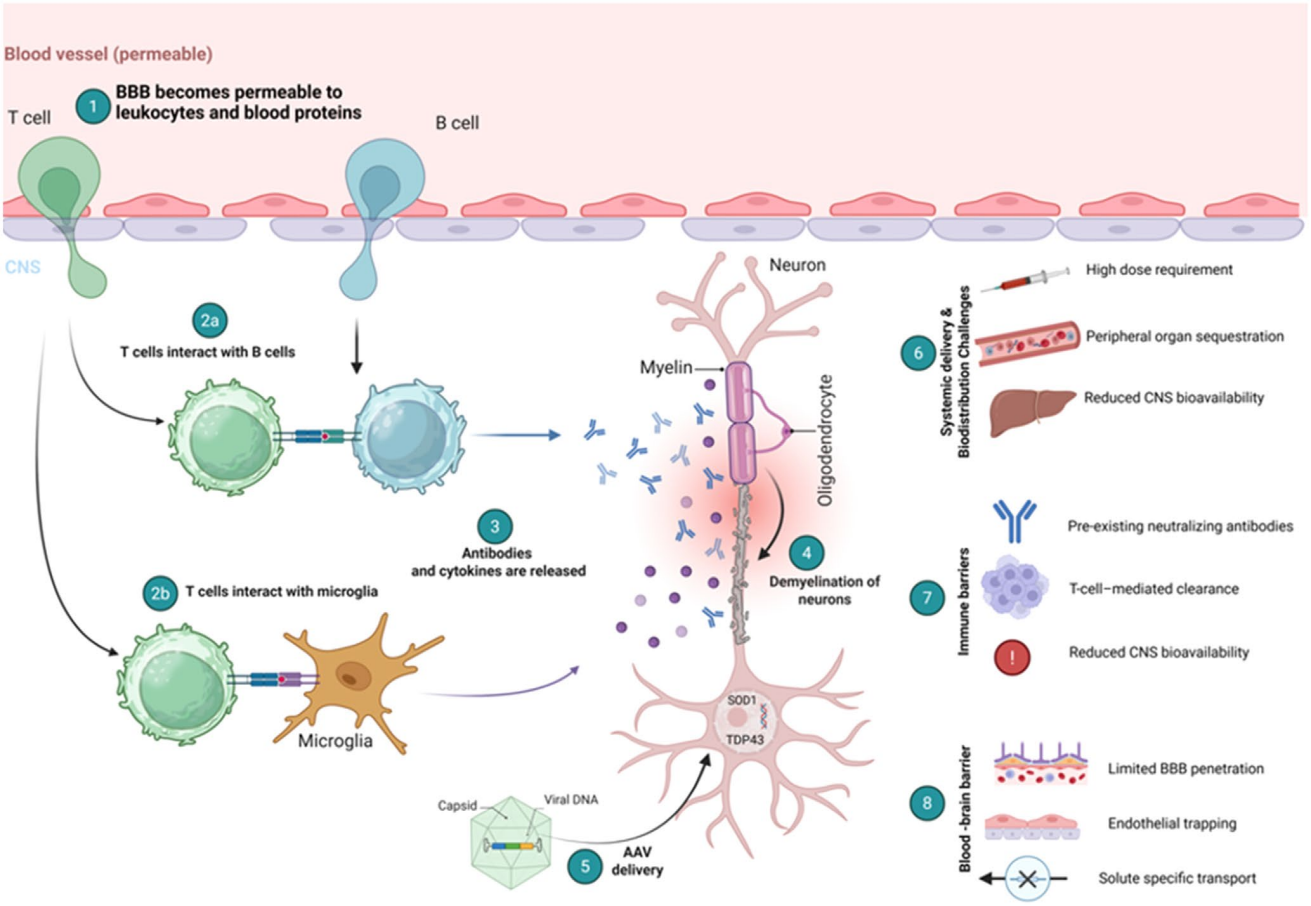
Blood–brain barrier (BBB)‐associated adeno‐associated virus (AAV) delivery challenges in amyotrophic lateral sclerosis (ALS). The topmost section depicts compromised blood‐vessel permeability during neuroinflammation, allowing T cells, B cells, and other components to cross the BBB and reach the CNS. (1) T‐cell infiltration and their interaction with B cells; (2a) microglia; (2b) triggering activation resulting in adaptive and innate immune responses against myelin antigen; (3) These activated cells secrete proinflammatory cytokines and antibodies and increase their trafficking around axons and oligodendrocytes, resulting in a chronic anti‐inflammatory milieu; (4) The events and mediators induce progressive injuries to oligodendrocytes and demyelination (loss of myelin sheath), eventually causing neuroaxonal degeneration and neurological dysfunction. (5) The central lower sections illustrate AAV carrying a therapeutic DNA that is intended to transduce glia and neurons to express neuroprotective genes or induce regeneration/restoration of axons and myelin sheath; (6) intravenous (i.v.) dosing requires high vector loads that limit CNS bioavailability and increase associated off‐target toxicity; (7) Pre‐existing neutralizing antibodies and cellular immune responses might destroy the AAV; (8) the BBB itself restricts AAV entry, posing a serious constraint in targeted delivery to neurons and oligodendrocytes. Recent AAV platforms are summarized in Table 1.

Immunogenic responses to therapeutic AAV administration can be mitigated through several strategies, including removal of neutralizing antibodies, the use of plasmapheresis, the development of engineered capsids with reduced immune recognition, and pre‐treatment with immunosuppressive agents. Together, these approaches aim to reduce vector clearance and enhance transduction efficiency. Current efforts are focused on refining these strategies, including transient modulation of immune checkpoints to facilitate the initial phase of AAV delivery. In parallel, optimization of expression systems has shown promise: for example, a hybrid promoter combining an AAV9 vector with a cytomegalovirus/chicken β‐actin (CAG) promoter has been shown to drive robust and widespread CNS transduction (Figure 8). Table 1 summarizes progress in AAV drug delivery across preclinical and human ALS clinical trials.

**TABLE 1:** Summary of AAV platforms summarizing advantages, limitations, CNS penetration efficiency, and translational status in preclinical models of ALS and in human trials.

Variant	Advantages	Limitations	CNS penetration efficiency	Translational status in ALS	References
AAV9	Highly robust, non‐pathogenic	High seroprevalence, Cardiotoxic, Hepatotoxic, and high systemic dosage is required	Moderate/low, capable of crossing the BBB	Preclinical/Early Clinical. Used in SOD1, SOD1‐G93A, and TDP‐43 rodent models and early phase of clinical trials	[46, 48, 50, 67]
AAVrh10	Rapid transgene expression, non‐pathogenic, and low seroprevalence	Peripheral organ toxicity and high systemic dosage are required	High and crosses the BBB effectively	Preclinical/Early Clinical. SOD1‐G93A rodent models and early phase of clinical trials	[68]
AAV‐PHP.B	Engineered for tissue tropism, Low organ toxicity	Species/strain specific	High penetration efficiency (50–100‐fold)	Preclinical TDP‐43 rodent models	[63]
AAV‐PHP.eB	Highly potent and low organ toxicity	Species/strain specific; Ly6a‐dependence	Ultra‐high in mice, moderate in rats	Preclinical	[69]
scAAV	Highly potent, Rapid transgene expression	Halved packaging capacity	Highly efficient/rapid onset	Preclinical, used in SOD1 rodent models	[70]
VTx‐002	Dual mechanism. Durable and highly selective	Vector‐induced immunogenic, phenotypic translational risk	Highly efficient	Clinical phase1/2, used for TDP‐43 proteinopathy	[71]

## 6 Advanced Drug Delivery in Preclinical Models of ALS

### 6.1 Novel Nanoparticle‐Based Edaravone Formulations to Improve Brain Bioavailability

To exert therapeutic effects in ALS, drugs must either cross the BBB or bypass it entirely [72]. The BBB is a highly selective, lipid‐rich interface formed by tight junctions between endothelial cells, which restricts the passage of most drugs and circulating molecules into the brain [73]. Although small lipophilic compounds may cross the barrier via passive diffusion, they remain susceptible to active efflux mechanisms that transport them back into the bloodstream [74].

**FIGURE 9:**
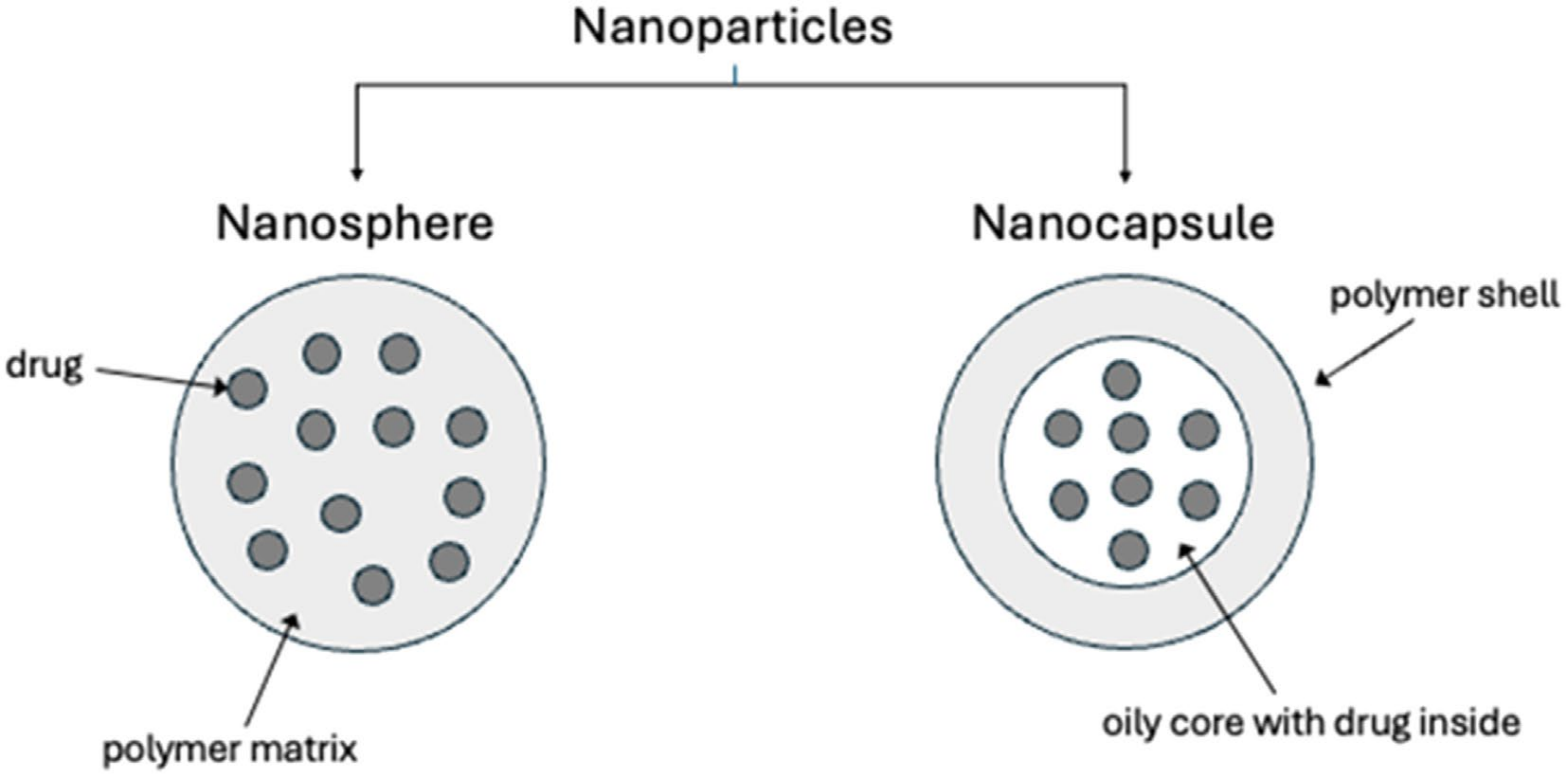
Nanocapsules and nanospheres are two types of nanoparticles. Nanospheres are polymeric matrices in which the drug is dispersed. Nanocapsules have a polymer‐based shell that encapsulates a core space containing the drug and solution.

One of the principal efflux transporters at the BBB is P‐gp, encoded by the *ABCB1* gene. This ATP‐dependent transporter, located on the luminal membrane of brain endothelial cells, plays a key protective role by exporting xenobiotics, toxins, and pharmacological agents, thereby limiting CNS drug penetration and posing a major challenge for CNS therapeutics [75]. Although invasive approaches can transiently increase BBB permeability, they carry significant risks, including infection, hemorrhage, and damage to surrounding brain tissue [76, 77]. In contrast, biodegradable polymeric nanoparticles (NPs) (Figure 9) represent a promising non‐invasive strategy for enhancing brain bioavailability. These approaches can be combined with intranasal or ‘nose‐to‐brain’ (N2B) delivery to bypass degradation and first‐pass metabolism associated with intravenous administration [72].

EDR (trade name Radicava) is an FDA‐approved treatment for ALS. In 2025, Aguiars et al. [78] developed EDR NPs using polymeric, lipidic, and lipid–polymer hybrid formulations. The aim was to produce NPs with a diameter of 100–300 nm, considered optimal for BBB penetration, with a monodisperse distribution and a negative zeta potential (< −30 mV). A negative zeta potential promotes electrostatic repulsion, reducing aggregation and limiting non‐specific interactions in vivo [79]. Polymeric and hybrid formulations of EDR showed no cytotoxicity in SH‐SY5Y cells, whereas lipid‐based carriers exhibited some toxicity, likely due to lipid peroxidation, along with reduced storage stability [78]. A lipid‐polymer hybrid nanoformulation of EDR in poly(lactic‐co‐glycolic acid) (PLGA) and D‐ɑ‐tocopheryl polyethylene glycol succinate demonstrated the highest level of radical scavenging (45.3% of ABTS^•+^ radical) of all EDR‐loaded NPs, compared with 67.7% for free EDR, still supporting the potential of NP‐based delivery to overcome limited CNS bioavailability.

**FIGURE 10:**
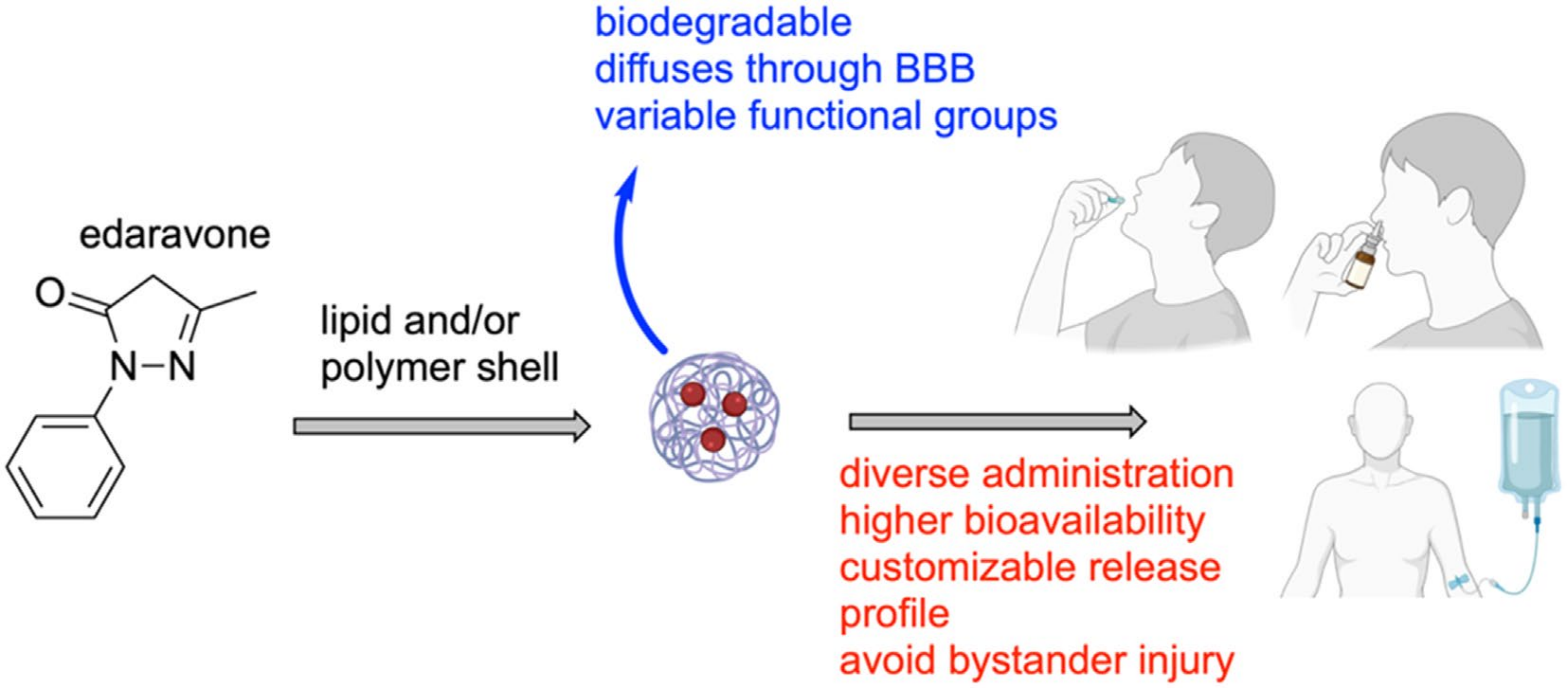
Edaravone (EDR) and edaravone‐loaded nanoparticles. Encapsulation of EDR within a lipid and/or polymer shell increases its bioavailability in the CNS by enhancing diffusion across the blood–brain barrier. This enables more diverse methods of administration, including oral and intranasal. Nanoformulations also offer other benefits, such as a tailorable release profile and the avoidance of injury to bystander organs through targeted diffusion in the body.

In 2023, similar PLGA‐based EDR‐loaded NPs (Figure 10) were developed, with diameters as small as ~90 nm and high stability (> 90% intact) for up to 30 days in a dark, cold, nitrogen‐protected environment [80]. This improved stability is attributed to the PLGA–polyethylene glycol (PEG) shell, which protects against light exposure and oxidative degradation [81]. It also enabled sustained drug release compared to free NP‐EDVs, exhibiting a slower release profile (50% release at 24 h) than NP‐EDVs, which released up to 80% in vitro in the presence of fetal bovine serum. Functionally, NP‐EDVs significantly reduced ROS levels in microglia‐like, BV‐2 cells, as demonstrated by decreased fluorescence in a dichlorofluorescein assay (*p* < 0.001), indicating antioxidant activity. When administered intranasally, NP‐EDVs showed minimal hepatic uptake compared to free EDR or intravenously administered NPs, suggesting reduced off‐target exposure and effective bypass of hepatic first‐pass metabolism [82].

Parikh et al. [83] proposed that lipid‐based NPs could bypass hepatic first‐pass metabolism via lymphatic uptake [84]. Both liquid lipid nanosystems (L‐LNS) and solid lipid nanosystems (S‐LNS) were developed, selected for optimal solubility in gastrointestinal fluids and reduced EDR glucuronidation. These systems increased oral bioavailability by 10.79‐fold (L‐LNS) and 9.29‐fold (S‐LNS), respectively, while enhancing dissolution rates. S‐LNS further demonstrated higher cellular uptake and improved neuroprotective effects in SH‐SY5Y cells compared to free EDR [83].

In 2022, EDV‐loaded chitosan NPs (ED‐CS‐NPs) intended for N2B delivery were synthesized [85]. Chitosan is a biodegradable and biocompatible polymer with low toxicity, making it suitable for controlled CNS drug delivery [86]. The optimized NPs had a size of 200 ± 0.273 nm and a zeta potential of −29 mV, consistent with efficient nasal delivery. In vitro, free EDR achieved only 21.32% diffusion after 3 h, whereas EDR‐CS‐NPs reached 91.80% release over the same period. Ex vivo studies using goat nasal mucosa showed 37.09% ± 0.24% drug diffusion after 3 h, along with sustained mucosal adhesion. Notably, higher chitosan content correlated with improved drug delivery performance, suggesting that polymer concentration plays a critical role in optimizing nasal NPs uptake.

### 6.2 Novel ALS Drug Formulations With Increased Bioavailability Across the BBB in Preclinical ALS Models

In 2020, Medina et al. [87] hypothesized that retinoid‐activating NPs could serve as a neuroprotective strategy for ALS. The retinoic acid signaling pathway has previously been implicated in ALS and other NDs [88]. The authors developed poly(lactic acid)‐poly(ethylene glycol) (PLA–PEG)‐based NPs of the retinoic acid receptor β agonist adapalene, thereby improving its aqueous solubility and enabling effective delivery. When administered intravenously three times per week in humanized SOD1^G93A^ mice, adapalene‐loaded NPs induced retinoid signaling within the CNS, delayed disease progression, and increased maximum lifespan (183 vs. 199 days). In addition to reducing motor impairment, treatment exerted neuroprotective effects and decreased expression of the neuroinflammatory marker Iba1 [89], supporting the combined therapeutic potential of retinoid pathway activation and NP‐based delivery.

miRNAs are small non‐coding RNAs known as critical regulators of neuronal development and motor function (REF). Dysregulation of specific miRNAs has been observed during ALS progression, making them attractive therapeutic targets. For example, SOD1^G93A^ mice exhibit upregulation of miRNA‐125b and miRNA‐128 and downregulation of miRNA‐124 and miRNA‐193b‐3p [90]. Niccolini et al. [91] explored graphene‐mediated miRNA delivery as a strategy for miRNA inhibition and replacement therapy in ALS [90]. Graphene consists of sp^2^‐hybridized carbon atoms arranged in a two‐dimensional honeycomb lattice, conferring unique mechanical and conductive properties. Graphene‐based materials offer a large surface area, relatively low toxicity [92], and the capacity to cross the BBB when reduced to nanoscale dimensions. Furthermore, their physicochemical properties and pharmacokinetics can be tailored through functionalization with biocompatible polymers or chemical groups.

**FIGURE 11:**
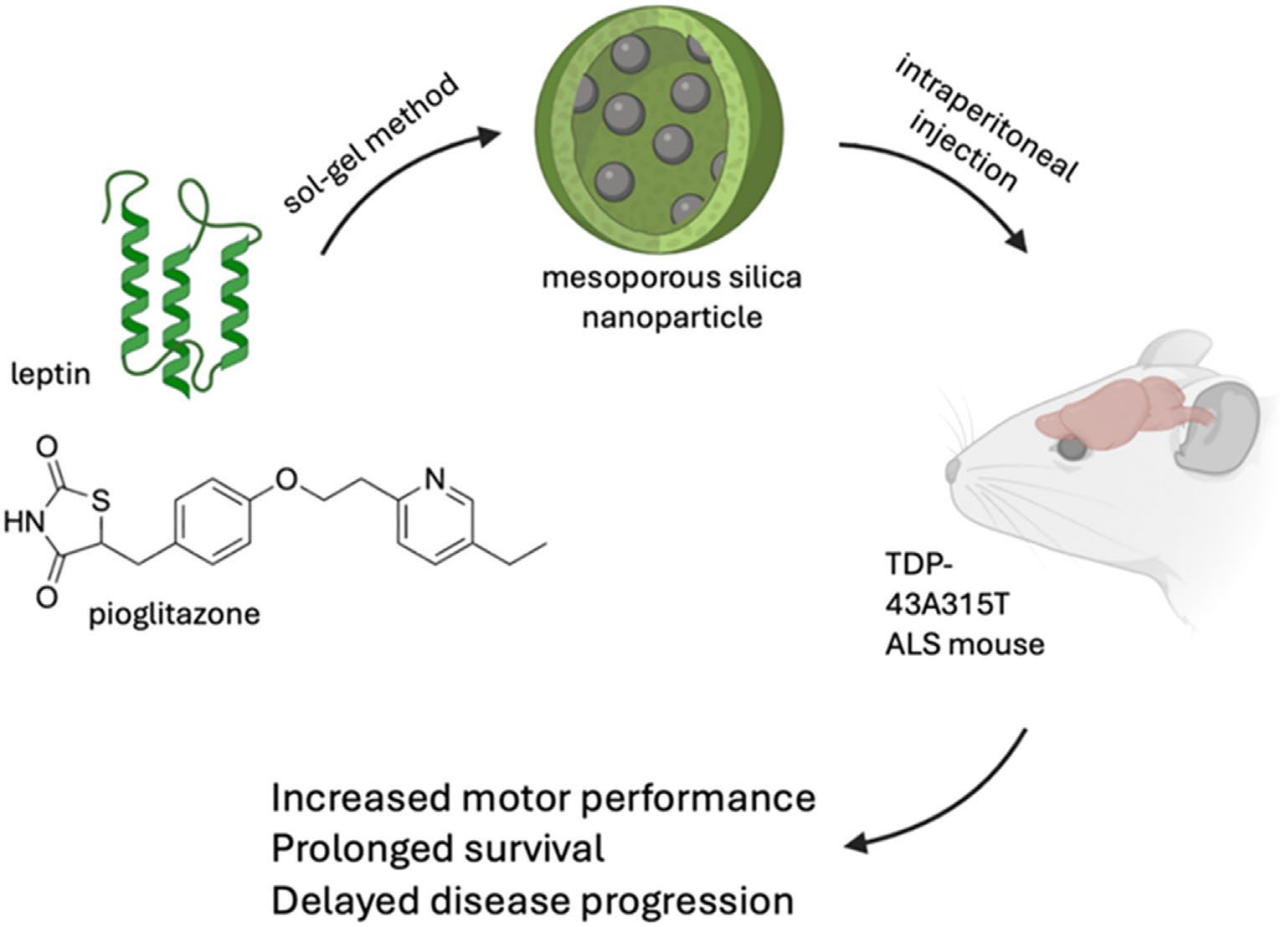
Description of leptin‐ and pioglitazone‐loaded mesoporous silica nanoparticles.

In 2022, Díaz‐García et al. [93] developed mesoporous silica NPs (MSNs) loaded with a therapeutic cocktail of leptin (LEP, a neuroprotective hormone) and pioglitazone (PIO, an anti‐inflammatory thiazolidinedione) (Figure 11). The MSNs were synthesized via a sol–gel process, functionalized with aminopropyl ligands, covalently conjugated with leptin, and loaded with pioglitazone. Comprehensive physicochemical characterizations (XRD, BET, TEM/SEM, NMR, FTIR, UV–Vis, CD spectroscopy, TGA) confirmed successful drug incorporation and structural integrity of the nanocarrier system (MSN‐LEP‐PIO). The NPs were almost spherical (~94 nm) and exhibited reduced surface area after drug loading, consistent with pore occupancy.

Drug‐release studies demonstrated that leptin was largely retained in the MSNs, with minimal release under physiological conditions, indicating that the system functions as a stable therapeutic entity rather than a conventional release platform. In contrast, pioglitazone exhibited a low but sustained release over 72 h, supporting its role in prolonged delivery. In vivo administration of MSN‐LEP‐PIO in TDP‐43 p.A315T ALS mice, via intraperitoneal injection, delayed disease progression, modestly extended survival, and significantly improved motor performance in rotarod testing at late disease stages. Silicon quantification confirmed the presence of NPs in both the motor cortex and spinal cord, indicating successful BBB penetration and CNS bioavailability.

This study represents one of the first demonstrations that MSNs can simultaneously deliver neuroprotective and anti‐inflammatory agents in ALS. The dual‐action combination of leptin + pioglitazone appeared to act synergistically to mitigate motor deficits, highlighting the potential of multi‐drug nanomedicine approaches in NDs. Importantly, the MSN‐LEP‐PIO system functions not only as a delivery vehicle but also as a multifunctional therapeutic platform, stabilizing its cargo and enabling efficient CNS targeting.

ALS still lacks an effective therapy capable of halting motor neuron loss. Ependymal stem/progenitor cells (epSPCs) in the ALS spinal cord are typically quiescent but can mount a compensatory proliferative response, suggesting that safely enhancing their self‐renewal may have therapeutic potential. FM19G11, a HIF‐pathway modulator, promotes epSPC expansion but is limited by poor aqueous solubility, restricting its delivery [94]. In 2019, Marcuzzo et al. [95] investigated whether mixed‐ligand gold NPs can improve intracellular delivery of FM19G11 and activate pro‐regenerative programs in epSPCs derived from SOD1^G93A^ ALS mice. epSPCs were isolated from SOD1^G93A^ and age‐matched B6.SJL mice controls at presymptomatic (8 weeks), onset (12 weeks), and late (18 weeks) stages and exposed for 24–48 h to vehicle, free FM19G11, or NP‐loaded FM19G11. NP uptake by neurospheres was confirmed using BODIPY‐labeled particles. FM19G11‐loaded nanoparticles increased epSPC abundance, most prominently at 12 weeks and to a lesser extent at 8 weeks, with minimal effects at 18 weeks. Markers of proliferative capacity and stemness were upregulated, including *Tert* transcripts (at 8–12 weeks) and Sox2/Oct4 at both mRNA and protein levels, peaking at 12 weeks. Pro‐survival signaling pathways were activated, with increased *Akt1*/*Akt3* transcripts and elevated phospho‐Akt protein. These changes were accompanied by mitochondrial remodeling, characterized by consistent upregulation of uncoupling protein 2 (*Ucp2*) (but not *Ucp1*), consistent with transient FM19G11‐induced uncoupling and a compensatory bioenergetic response. At the post‐transcriptional level, miR‐19a expression increased, while its target PTEN decreased at both mRNA and protein levels at 12 weeks, supporting activation of the PI3K/AKT pathway. Importantly, NPs alone did not enhance proliferation or induce detectable nanotoxicity; the observed effects were dependent on FM19G11 loading.

These findings delineate a mechanistic cascade linking NP‐enabled drug delivery to mitochondrial adaptation (via UCP2), suppression of the miR‐19a/PTEN axis, activation of AKT signaling, and induction of stemness‐associated genes, ultimately promoting epSPC self‐renewal. Two translational implications emerge: first, therapeutic timing appears critical, with maximal efficacy observed near symptom onset; and second, NPs function as delivery vehicles rather than independent therapeutic agents. Limitations include the study's in vitro nature and reliance on a familial SOD1 model. Future work should focus on in vivo validation, including dosing, biodistribution, functional outcomes, safety, and extension to sporadic ALS. Nevertheless, FM19G11‐loaded gold NPs warrant further preclinical development, particularly targeting early disease stages.

In a complementary approach, DeCoteau and team in 2016 [96], developed ultrasmall cerium oxide nanoparticles (CeNPs; ~3.3 nm) as catalytic antioxidants, stabilized with citrate and ethylenediaminetetraacetic acid (EDTA). CeNPs were administered at 20 mg/kg twice a week, with treatment initiated at the onset of measurable motor weakness, as assessed by the hanging‐wire test. Behavioral and survival analyses were conducted in a blinded manner, and cerium biodistribution was quantified using inductively coupled plasma mass spectrometry (ICP‐MS). CeNP treatment resulted in significant therapeutic effects. Treated mice exhibited a slower decline in motor function during the first 25 days, with better preservation of grip strength and endurance compared to vehicle‐treated controls. The interval between initial weakness and progression to severe impairment (score < 5) was prolonged, indicating preservation of a functional pre‐symptomatic window. Survival analysis demonstrated a statistically significant benefit, with median survival of 33.0 ± 3.7 days in treated mice versus 22.0 ± 2.5 days in controls (*p* = 0.022), with consistent effects across sexes. Biodistribution studies confirmed the presence of cerium within the CNS, albeit at lower concentrations than in the liver and spleen, where accumulation was highest; nonetheless, CNS detection supports partial BBB penetration or evasion.

**FIGURE 12:**
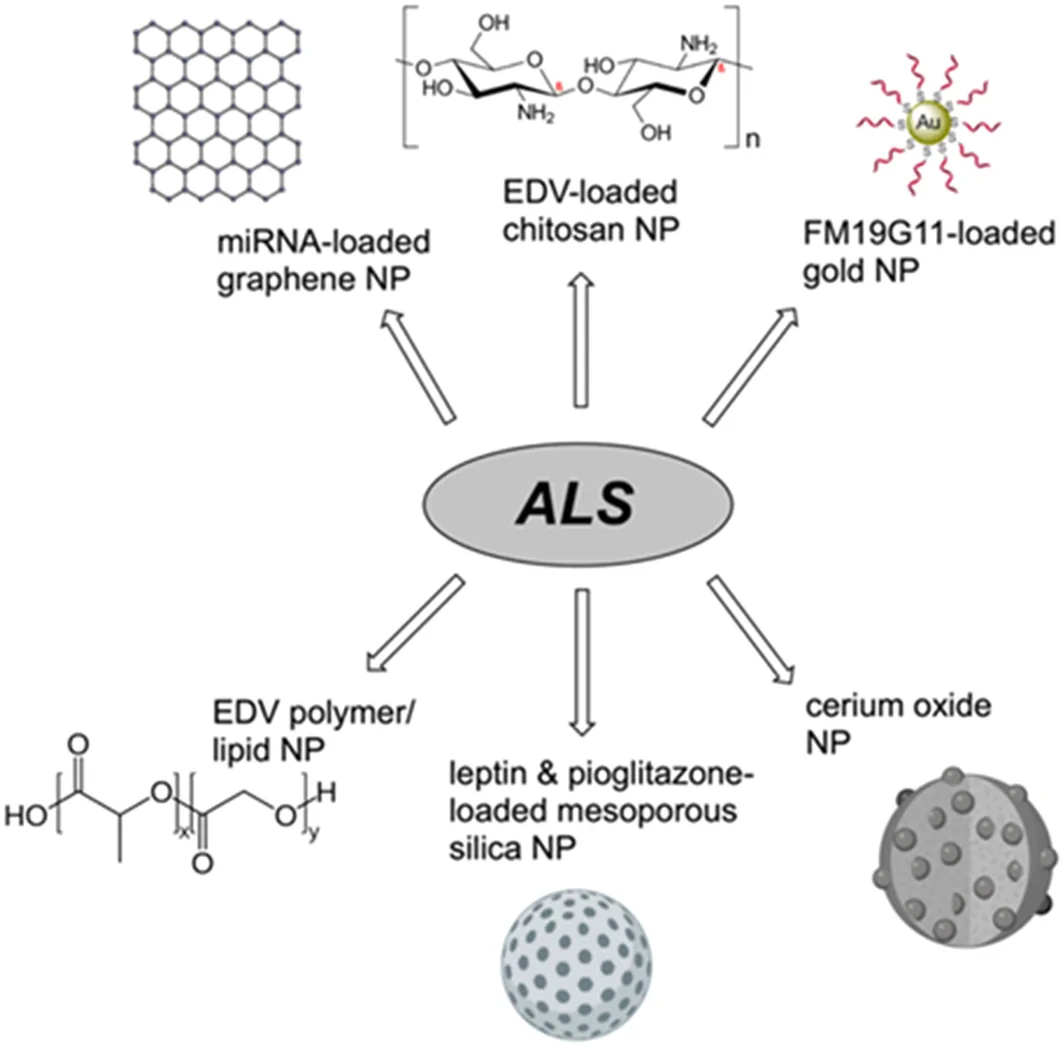
Various formulations for ALS treatment. These include antioxidant cerium oxide, various edaravone (EDR)‐loaded nanoformulations, and miRNA‐therapy‐based delivery systems. These nanoformulations may be used to treat oxidative stress, gene mutations, or stem/progenitor cells in ALS models.

Catalytic antioxidant NPs such as CeNPs may delay ALS progression even when administered after symptom onset. Rather than preventing disease initiation, they appear to stabilize neuronal function during a critical window of vulnerability, thereby prolonging motor neuron survival. Their capacity for catalytic redox cycling and prolonged tissue residence may overcome limitations associated with conventional small‐molecule antioxidants (Figure 12). Table 2 summarizes progress in advanced nanoparticle‐based drug delivery across preclinical and human ALS clinical trials.

**TABLE 2:** Summary of nanoparticle platforms summarizing therapeutic outcomes, limitations, CNS penetration efficiency, and translational status in preclinical models of ALS and in human trials.

Nanoparticle type and size	Active ingredient	Therapeutic outcome	CNS penetration efficacy	Translational status in ALS	References
Polymeric, hybrid, or lipidic NPs 100–300 nM −30 mV zeta potential	Edaravone	No cytotoxicity in human‐derived neuroblastoma SH‐SY5Y cells (polymer & hybrid), moderate toxicity from lipidic NPs	Untested, but within the optimal size range for BBB penetration	Pre‐clinical	[78]
Poly(lactic‐co‐glycolic acid) (PLGA)‐based NPs ~90 nM	Edaravone	Reduced H_2_O_2_‐induced oxidative stress in murine microglial cells (BV‐2)	Optical imaging following intravenous and intranasal administration in mice confirm uptake in the CNS	Pre‐clinical	[80]
Solid and liquid lipid nanosystem < 100 nM	Edaravone	Increased cellular uptake and neuroprotective effects in SH‐SY5Y cells Significant improvement in oral bioavailability in rats	Untested, but within the optimal size range for BBB penetration	Pre‐clinical	[83]
Chitosan‐based nanospheres and nanocapsules 200 nM −29 mV zeta potential	Edaravone	91.80% drug diffusion in 3 h (21.32% for free Edaravone)	Untested, but within the optimal size range for BBB penetration	Pre‐clinical	[85]
Poly(lactic acid)‐poly(ethylene glycol) (PLA–PEG)‐based NPs	Adapalene	Delayed disease progression and improved lifespan in SOD1‐G93A mice	Authors proposed that adapalene transfers from NPs to BBB endothelial cells, rather than direct NP passage	Pre‐clinical, tested in SOD1‐G93A mice	[87]
Mesoporous silica nanoparticles 94 ± 15 nM	Leptin and pioglitazone	Delayed disease progression and improved lifespan in TDP‐43 p.A315T mice	Silicon quantification confirmed NP presence in motor cortex and spinal cord	Pre‐clinical, tested in TDP‐43 p.A315T mice	[93]
Gold NPs ~4.5 nM	FM19G11 (a hypoxia‐inducible factor modulator)	Upregulated cellular pathways of self‐renewal and proliferation in SOD1 G93A ependymal stem progenitor cells (epSPCs)	Untested, but within the optimal size range for BBB penetration	Pre‐clinical, tested in epSPCs of SOD1 G93A mice	[95]
Cerium oxide NPs ~3.3 nM	Cerium oxide as a catalytic antioxidant	Delayed disease progression and improved lifespan in SOD1 G93A mice	Transmission electron microscopy and tissue redox activity indicate BBB passage	Pre‐clinical, tested in SOD1 G93A mice	[96]
Lipid NP system modified with cRGD peptides ~93 nM	Edaravone and Kaempferol	Delayed disease progression and improved lifespan in SOD1 G93A mice	In vivo and ex vivo imaging indicated elevated NP levels in cerebral tissue	Pre‐clinical, tested in SOD1 G93A mice	[97]
Lipid NP system 20–40 nM	GM1 ganglioside	Improved motor coordination and lifespan in SOD1 G93A and C9‐500 (*C9orf72* mutation) mice models	NPs exhibited two‐fold increase in brain GM1 levels compared to free GM1 administration	Pre‐clinical/early clinical, tested in SOD1 G93A and C9‐500 mice models Long‐term patient toxicity and Phase 1 Parkinson's trials in progress	[98]
Gold nanocrystal suspension 20–45 nM	Gold nanocrystal suspension with protein corona including significant apolipoprotein content	Positive biomarker data and reduced risk of death at 30 mg dosing	Pre‐clinical and clinical studies indicate ability to cross BBB successfully	Completed phase 2 RESCUE‐ALS, (NCT04098406) trial and phase 3 (HEALEY) platform trials, (NCT04297683), failing to meet its primary functional endpoint. However, exploratory data and open‐label extensions showed a strong association with significantly prolonged survival. Confirmatory phase 3 trial (RESTORE‐ALS) planned	[99, 100, 101]

## 7 Current Shift Toward Antisense Oligonucleotide (ASO)‐Based Therapeutics and Their Limitations

Further advances in gene‐silencing therapies have led to the development of antisense oligonucleotides (ASOs) for the treatment of NDDs. In April 2023, the FDA granted accelerated approval to tofersen (BIIB067; Qalsody), the first ASO therapy approved for superoxide dismutase 1‐associated ALS (SOD1‐ALS). Tofersen is designed to bind SOD1 messenger RNA (mRNA), promoting its degradation and consequently reducing the synthesis and aggregation of the toxic SOD1 protein [102, 103]. Approval was primarily supported by reductions in neurofilament light chain (NfL), a nonspecific surrogate biomarker of neuroaxonal injury [104]. However, despite demonstrating target engagement and biomarker improvement, definitive evidence that tofersen slows clinical disease progression remains limited, and concerns regarding its overall benefit–risk profile have been raised following recent clinical studies [103, 105]. Furthermore, owing to its limited ability to cross the BBB, tofersen requires repeated intrathecal administration, which may pose challenges for long‐term patient adherence and clinical implementation [106] when compared with orally or intravenously administered small‐molecule therapies such as RLZ and EDR.

The therapeutic potential of ASOs has expanded considerably across NDDs. Beyond ALS, several ASO‐based therapies are being investigated for disorders characterized by toxic protein accumulation. For example, BIIB080 (MAPTRx; ISIS 814907), an ASO targeting microtubule‐associated protein tau (MAPT) mRNA, has demonstrated dose‐dependent reductions in CSF tau concentrations in patients with AD, providing clinical proof of concept for tau‐lowering strategies [107]. Similarly, ASOs targeting leucine‐rich repeat kinase 2 (LRRK2) are currently under clinical evaluation for Parkinson's disease. These advances highlight the growing promise of gene‐silencing therapeutics as a precision medicine approach for NDDs [107].

Despite these encouraging developments, several fundamental challenges continue to limit the broader clinical translation of ASO‐based therapies [108, 109, 110]. First, sequence‐dependent and sequence‐independent off‐target interactions remain a significant concern, as ASOs can inadvertently bind non‐target RNA and DNA molecules, potentially leading to unintended biological effects [111]. Second, long‐term safety remains incompletely characterized, necessitating extensive validation in both preclinical and clinical settings to better define toxicological profiles and treatment‐associated adverse events [112, 113]. Third, ASOs can elicit innate and adaptive immune responses, creating additional challenges related to tolerability and immunogenicity [114, 115].

However, perhaps the greatest obstacle to the widespread implementation of ASO therapeutics for NDDs is efficient CNS delivery. Due to their large molecular size, polyanionic nature, and poor membrane permeability, ASOs exhibit limited penetration across the BBB [116, 117]. Following systemic administration, they preferentially accumulate in peripheral organs such as the liver and kidneys, resulting in suboptimal CNS exposure [118]. Furthermore, intracellular trafficking barriers, including sequestration within endosomal and lysosomal compartments, can substantially reduce the fraction of ASOs reaching their intended intracellular targets [119]. Consequently, achieving sufficient target engagement often requires direct administration into the cerebrospinal fluid or brain parenchyma through invasive procedures such as intrathecal or intracerebroventricular injection [118].

**FIGURE 13:**
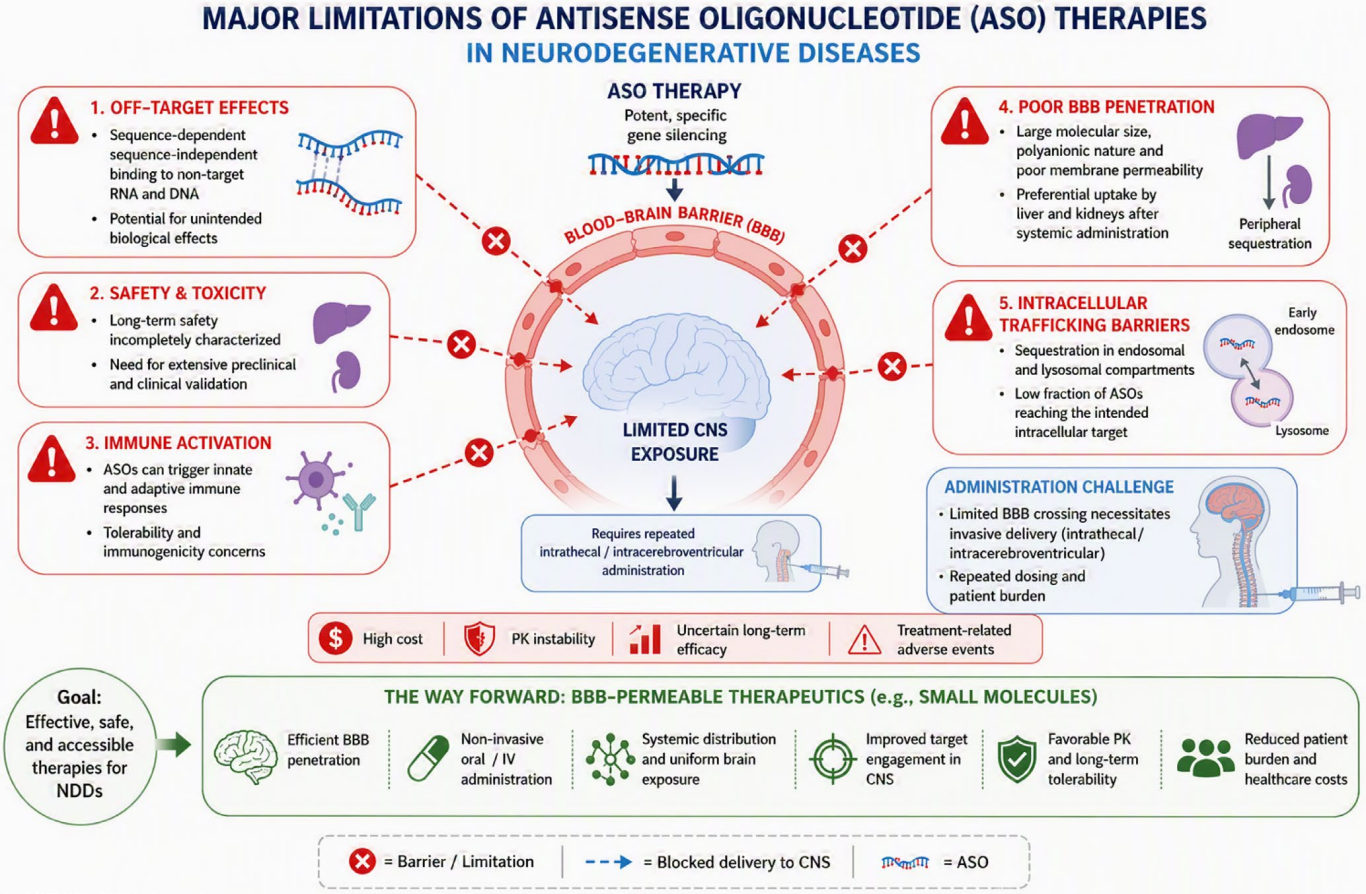
Limitations of antisense oligonucleotide (ASO) therapies for neurodegenerative diseases (NDDs). Despite their ability to achieve selective gene silencing, ASOs face important translational challenges, including off‐target effects, immunogenicity, safety concerns, poor blood–brain barrier (BBB) penetration, and intracellular trafficking barriers. These limitations result in limited central nervous system exposure, the need for repeated intrathecal administration, and increased treatment burden, highlighting the need for safe, non‐invasive, BBB‐permeable therapeutic approaches.

Collectively, these limitations underscore a central paradox in ASO therapeutics: while advances in molecular design have markedly improved target specificity and gene‐silencing efficacy, delivery remains the principal bottleneck preventing their full clinical potential. Therefore, the development of safe, non‐invasive, and BBB‐permeable delivery platforms capable of improving CNS biodistribution, intracellular trafficking, and long‐term stability will be critical for realizing the next generation of ASO therapies for NDDs [106, 120]. More broadly, persistent challenges, including PK instability, uncertain long‐term efficacy, treatment‐related adverse events, and high healthcare costs, continue to contribute to the substantial translational gap between experimental success and meaningful clinical benefit in patients with NDDs. Furthermore, it is important to accelerate the discovery and development of small molecules that can cross the BBB and offer significant delivery and PK advantages over ASOs. Specifically, their lower molecular weight and lipophilicity enable non‐invasive oral or intravenous administration, systemic distribution, and deeper, more uniform penetration into the brain, thereby reducing key limitations such as uneven CNS distribution and patient burden [2, 121, 122] (Figure 13).

## 8 Discussion

### 8.1 Comparative Evaluation of Approaches With Current Translational Promise

Therapeutic strategies for ALS can be broadly categorized into disease‐modifying interventions and technologies that ameliorate CNS drug delivery. Among current approaches, ASOs have achieved the greatest translational success. The approval of tofersen for SOD1‐associated ALS provides proof‐of‐concept that genetically targeted therapies can modify disease‐relevant biology in ALS patients. This success is likely attributable to three factors: a clearly defined molecular target, the ability to directly engage disease‐causing transcripts with high specificity, and an established intrathecal delivery route that circumvents the BBB. Importantly, clinical studies have demonstrated target engagement, including reductions in SOD1 protein levels and NFL, a surrogate biomarker of neuroaxonal injury [123].

In contrast, AAV‐based gene therapies remain constrained by substantial translational challenges despite their theoretical appeal as potentially curative, single‐administration treatments. Systemic delivery is limited by inefficient BBB penetration, often necessitating high viral doses that increase the risk of off‐target toxicity. Furthermore, pre‐existing and treatment‐induced immune responses can significantly reduce transduction efficiency and compromise therapeutic durability. A critical unresolved limitation is the lack of a clinically practical mechanism to terminate transgene expression in the event of toxicity, raising important safety considerations for irreversible gene‐modifying interventions [124]. Consequently, although AAV platforms have demonstrated remarkable efficacy in preclinical models of ALS, their path to broad clinical implementation in ALS remains uncertain.

Small‐molecule therapeutics represent a potentially more scalable and patient‐friendly alternative. Historically, however, most organic small molecules have failed in complex diseases like ALS because of inadequate PK properties, poor CNS exposure, and limited target engagement. Advances in medicinal chemistry may offer a route to overcome these limitations. Further, rational prodrug design can improve BBB permeability and metabolic stability, while multi‐target‐directed ligands (MTDLs) may be particularly attractive in ALS, where neuroinflammation, oxidative stress, mitochondrial dysfunction, and protein misfolding converge to drive multipathological disease progression. Given the multifactorial nature of ALS, molecules that target multiple pathways and modulate multiple pathological processes simultaneously may ultimately prove more effective than highly selective single‐target approaches [125, 126, 127].

Within this framework, advanced drug delivery approaches, including nanomedicine and FUS, should be viewed primarily as enabling technologies rather than stand‐alone therapies. Both approaches seek to address one of the most pressing barriers in ALS drug development: inadequate CNS drug exposure. Nanoparticle formulations can improve drug stability, circulation time, and tissue distribution, whereas FUS enables transient, spatially controlled opening of the BBB, thereby facilitating delivery of impermeable therapeutics. However, neither platform directly addresses the underlying biology of ALS; their clinical value will ultimately depend on the efficacy of the therapeutic cargo they deliver.

Taken together, ASOs currently represent the most clinically mature precision‐medicine strategy for ALS. Nevertheless, their reliance on repeated intrathecal administration limits scalability and broad applicability. Over the longer term, the greatest therapeutic impact may arise from the convergence of medicinal chemistry, BBB‐aware drug design, and advanced delivery technologies. The development of orally bioavailable, CNS‐penetrant, multi‐target small molecules, potentially augmented by nanoparticle or ultrasound‐assisted delivery, offers a compelling path toward reducing patient burden while expanding treatment access across the heterogeneous ALS population.

### 8.2 Addressing the Translational Bottleneck: Key Unanswered Questions and Future Priorities for BBB/BSCB‐Targeted Therapies in ALS

A critical obstacle to developing effective disease‐modifying therapies for ALS is the poorly understood operational timeline of BBB and BSCB disruption. It remains highly contested whether the observed barrier hyperpermeability and structural degeneration represent a primary pathogenic driver that accelerates motor neuron death or merely a secondary byproduct of chronic, systemic neuroinflammation [128]. Resolving this cause‐versus‐consequence dilemma is essential; without defining the precise molecular cascades that govern tight junction disassembly and the concurrent upregulation of efflux transporters such as P‐glycoprotein, therapeutic strategies will continue to fail due to unrecognized, disease‐specific pharmacoresistance [129].

**FIGURE 14:**
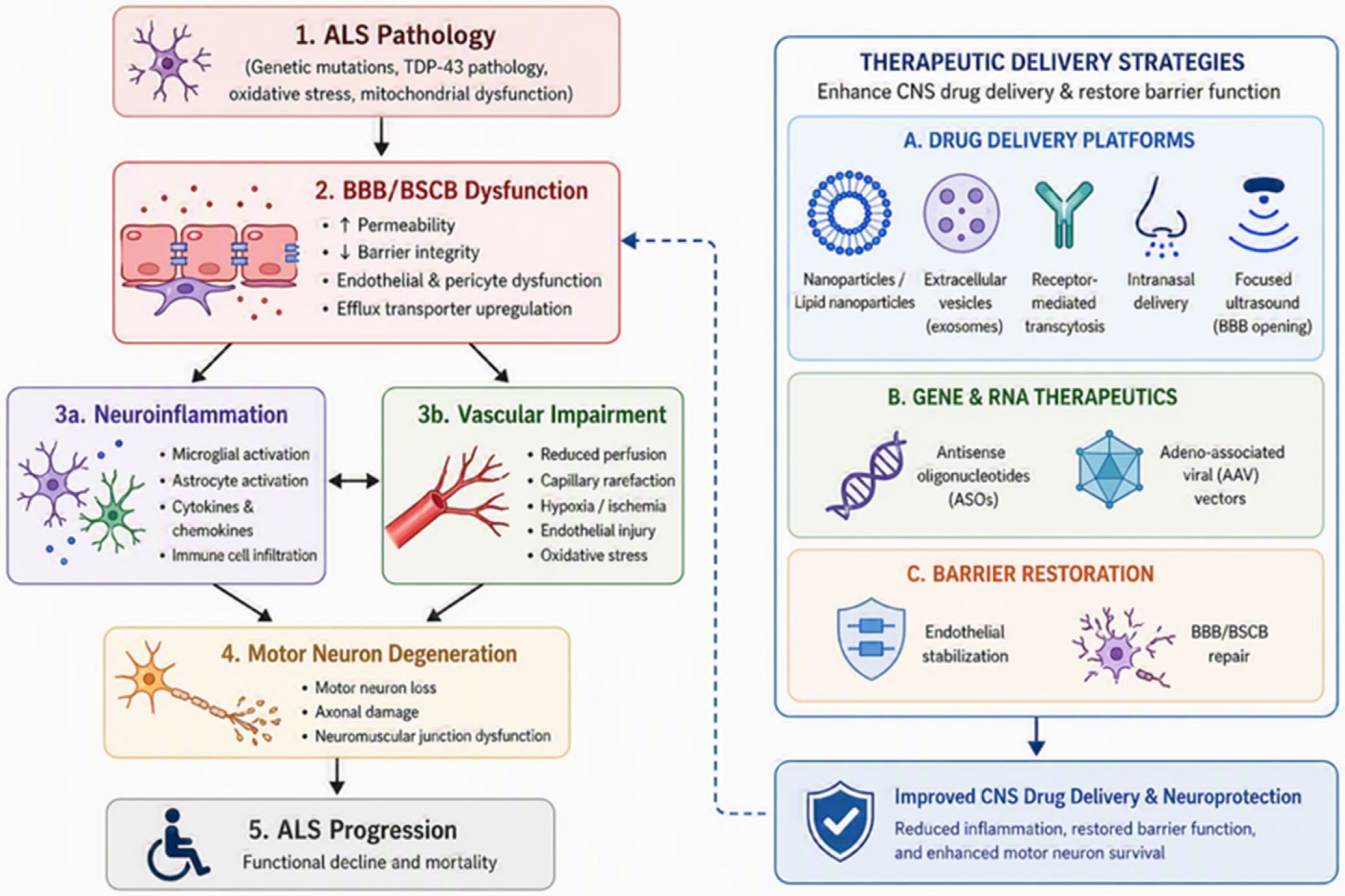
Summary of schematic integrating BBB/BSCB dysfunction, neuroinflammation, vascular impairment, and therapeutic delivery strategies into a unified ALS disease framework. Therapeutic delivery approaches, including nanoparticles, extracellular vesicles, ASOs, AAV vectors, focused ultrasound, and barrier‐restorative therapies, may improve CNS drug delivery and neuroprotection.

Furthermore, realigning translational priorities requires a candid re‐evaluation of current CNS drug delivery strategies. Although macromolecular approaches such as ASOs have attracted intense focus, their high molecular weight limits passive barrier penetration, requires invasive intrathecal delivery, and often leads to poor parenchymal distribution [130]. To bypass these logistical and biological bottlenecks, the next generation of ALS drug discovery must decisively pivot toward small molecules utilizing various medicinal chemistry approaches. Small molecules possess a fundamentally superior structural aptitude for non‐invasive, passive diffusion across the BBB/BSCB. Further, (Figure 14) summarizes how blood–CNS barrier dysfunction links neuroinflammation, vascular pathology, and motor neuron degeneration in ALS while simultaneously limiting therapeutic access to the CNS. Therefore, next‐generation ALS therapeutics should be designed with BBB permeability, optimal CNS exposure, high brain‐to‐plasma ratios, and minimal efflux liability as key development criteria, alongside emerging delivery strategies such as ASOs, AAV vectors, nanoparticles, and extracellular vesicles [131, 132].

## 9 Conclusion

In conclusion, despite 150 years of research since ALS was first described, the disease remains uniformly fatal, with no curative or robust disease‐modifying therapies successfully translating to patient benefit. A growing body of evidence suggests that dysfunction of the BBB and BSCB plays a critical role in this translational failure, not only by restricting xenobiotic transport into the CNS but also by creating a dynamic and compensatory barrier environment that continues to limit effective drug delivery even as barrier integrity deteriorates during disease progression. Consequently, many neuroprotective agents demonstrate efficacy in vitro yet fail in clinical trials due to poor CNS bioavailability and non‐ideal CNS drug‐like properties. Addressing this challenge will require longitudinal assessment of BBB and BSCB integrity through robust biomarker development, alongside the implementation of advanced, non‐invasive delivery strategies to enhance CNS penetration. Further, applying medicinal chemistry approaches to design drugs that could exhibit ideal CNS drug‐like properties, permeate the BBB from a very early stage of the disease, preserve its integrity, and demonstrate neuroprotective effects. Such approaches are essential for bridging the gap between experimental neuroprotection and meaningful therapeutic outcomes for ALS patients.

## Author Contributions

All authors read and approved the final manuscript. Nitesh Sanghai, K.P., Nidhi Sharma, S.L., T.C.Y., P.M., D.A., S.P., and P.C.M. contributed to the concept and participated in the literature review, initial drafting, referencing, and editing of the manuscript. G.K.T. oversaw the writing, review, and editing process. Further, Nitesh Sanghai collaborated and communicated with the manuscript's collaborators at every step of development. All authors have reviewed and approved the final version of the manuscript. G.K.T. also secured funding for the project.

## Funding

This research was supported by the Natural Sciences and Engineering Research Council of Canada (NSERC) Discovery Grant (grant number RGPIN‐2017‐05938) and the Canadian Institutes of Health Research (CIHR) grant (grant number 202210PJT‐495295) to G.K.T.

## Conflicts of Interest

The authors declare no conflicts of interest.

## Data Availability

Data sharing not applicable to this article as no datasets were generated or analysed during the current study.
